# Pan-genome analysis and ancestral state reconstruction of class halobacteria: probability of a new super-order

**DOI:** 10.1038/s41598-020-77723-6

**Published:** 2020-12-03

**Authors:** Sonam Gaba, Abha Kumari, Marnix Medema, Rajeev Kaushik

**Affiliations:** 1 Division of Microbiology, ICAR-Indian Agricultural Research Institute, New Delhi, India; 2 Amity Institute of Biotechnology, Amity University, Noida, Uttar Pradesh India; 3 Bioinformatics Group, Wageningen University, Wageningen, The Netherlands

**Keywords:** Data acquisition, Data mining, Phylogeny, Archaeal genomics

## Abstract

Halobacteria, a class of Euryarchaeota are extremely halophilic archaea that can adapt to a wide range of salt concentration generally from 10% NaCl to saturated salt concentration of 32% NaCl. It consists of the orders: Halobacteriales, Haloferaciales and Natriabales. Pan-genome analysis of class Halobacteria was done to explore the core (300) and variable components (Softcore: 998, Cloud:36531, Shell:11784). The core component revealed genes of replication, transcription, translation and repair, whereas the variable component had a major portion of environmental information processing. The pan-gene matrix was mapped onto the core-gene tree to find the ancestral (44.8%) and derived genes (55.1%) of the Last Common Ancestor of Halobacteria. A High percentage of derived genes along with presence of transformation and conjugation genes indicate the occurrence of horizontal gene transfer during the evolution of Halobacteria. A Core and pan-gene tree were also constructed to infer a phylogeny which implicated on the new super-order comprising of Natrialbales and Halobacteriales.

## Introduction

Halobacteria^1,2^ is a class of phylum Euryarchaeota^3^ consisting of extremely halophilic archaea found till date and contains three orders namely Halobacteriales^4,5^ Haloferacales^5^ and Natrialbales^5^. These microorganisms are able to dwell at wide range of salt concentration generally from 10% NaCl to saturated salt concentration of 32% NaCl^6^. Halobacteria, as the name suggests were once considered a part of a domain "Bacteria" but with the discovery of the third domain "Archaea" by Carl Woese et al.^7^, it became part of Archaea. Therefore, these microorganisms are alternatively called as haloarchaea isolated from salt marshes, subterranean and surface salt lakes, salt domes prevailing under the sea and also in halite deposits resulted from the evaporation of ancient seas from different parts of the world^8^. The first one being isolated from Permian Salt Sediment in 1963^9^ and since then they have gained lot of attention because of their obvious feature to withstand extreme environments and the need to find the mechanisms underlying this adaptation. Moreover, their isolation from rock salts deposited over millions of years, has proved its remarkable property of longevity surviving high UV radiation, high temperature and salinity. This has also led to the hypothesis of their existence in extra-terrestrial habitats^8–12^.

Despite the known advantageous characteristics of class Halobacteria, the genetic repertoire inferring them wide adaptability to harsh conditions is still under investigation. Although, a recent study has focussed on core-genes of class Halobacteria but the genetic diversity embedded in the class was under-represented^13^. The genetic repertoire can be of great use to recuperate barren saline lands by transferring of important genes to agricultural crops. Some of the halobacterial species are accustomed to adapt to high concentration of toxic halogenated organic compounds present in agricultural biocides. They also have high resistance to harsh and damaging UV radiation which is a major environmental concern in the current world. Therefore, these microorganisms have a great potential in agricultural studies and this delineates the need to investigate their genomes and to find the diversity embedded in this class.

Though, there has been a vast increase in genomic data (139 genomes) of halobacterial species in recent years credited to the low cost of next generation sequencing and readily available tools for assembly and annotation but a clear division of organisms is cumbersome and therefore, taxonomic identification of novel organisms is difficult. There also have been reports indicating divergent copies of 16s rRNA in some species of Halobacteria having ~ 5% divergence and the 16s ribosomal RNA of some species of Halobacteria have sequence similarity of 83.2%^1,14^. Therefore, using a single gene like 16s ribosomal RNA has a low discriminatory power and so not a reliable method to infer the phylogeny. In contrast to this, multi-gene phylogeny for example core genes will be a better strategy to correctly infer its phylogeny. However, the importance of pan-genomic tree cannot be underestimated as this gives the snapshot of presence and absence of gene families. Thus, a strategy encompassing all the genomes available in databases to find important genomic characteristics can be fruitful. One such strategy is Pan-genomics.

A pangenome is a collection of all the genomic features present in a group of organisms including the core which is defined as the genomic features present in all the organisms of the group. Though core can give the group specific features, accessory component is also as important as core to reveal the genetic diversity present among the group specially when the taxonomic resolution is as high as class. A pan-genomic matrix along with core-gene tree can also give important information about the last common ancestor of the class and the process of evolution in terms of genome expansion or genome reduction.

In present study, we used 111 genomes of halobacterial species present in the database at the time of data gathering, selected on the basis of completeness and contamination of the genomes for creating pan Halobacteria and to study the genetic diversity present among the members of the class. Two genomes of each class of phylum Euryarchaeota were also taken in the study considering them as close relatives of Halobacteria. Functional annotation of core and non-core genes gave information about group specific and diverse genes respectively. Both core and pan-genomic tree were constructed to infer the correct phylogeny of the class. Although there are studies focussing on the evolution of archaea and discussing about haloarchaea as the branch of the archaeal phylogenetic tree having highest number of horizontal gene transfers from eubacteria, a full-fledged study on identifying the halobacterial last common ancestor is lacking^15–17^. A very recent study also focussed on the novel chimeric genes formed from eubacterial components and enriched in carbohydrate metabolism^18^ but this is the first study to present ancestral state reconstruction along with gene gain loss information embedded in the class Halobacteria which is important to get the insights into its evolution.

## Results

### The halobacterial dataset

A halobacterial set of non-redundant 139 genomes spanning three families Halobacteriales^4^, Haloferacales^5^ and Natrialbales^5^ available at that time was considered for our analysis. The genome size ranges from 596,275 base pairs to 6,839,548 base pairs, GC content from 0.47 to 0.70, completeness from striking low 9% to 100% and contamination as low as 0% to 24% being the highest. (Supplementary Table S1). The Euryarchaeota set comprised of 408 non-redundant set of genomes. Its genome size ranges from 512,945 base pairs to 6,839,548 base pairs and GC content from 0.24 to 0.70 (Supplementary Table S2). Supplementary Table S2 also presents completeness and contamination of genomes.

**Figure 1:**
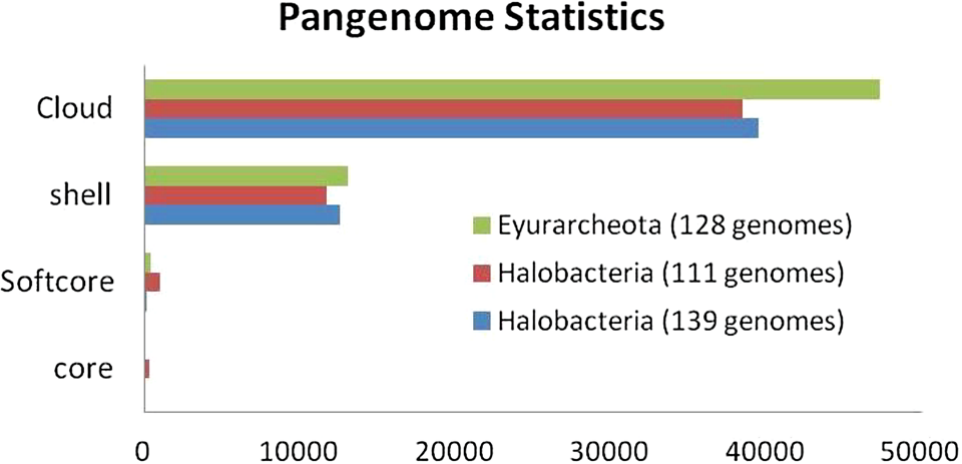
Graph showing Core, Softcore, Shell and Cloud clusters of the pan Halobacteria (139 genomes), pan Halobacteria (111 genomes) and pan Euryarchaeota (128 genomes).

A total of 111 halobacterial genomes out of 139 were selected on the basis of completeness (> 99%) and contamination (< 5%) (Supplementary Table S3). The Euryarchaeota dataset was also filtered to get the complete genomes but only 2 genomes of each class except Halobacteria having more than 99% completeness and contamination less than 5 percent were considered. (Table 1) as described in the methodology section. However, Nanohaloarchaea^19^ class had only 2 genomes (*Candidatus Nanosalinarum* sp. J07AB56 and *Candidatus Nanosalina* sp. J07AB43)^19^ at that time having completeness of 78.5% and 73.5% but still added in the analysis to get the full phylogenetic tree for the phylum Euryarchaeota^3^. Also, only one organism belonged to class Methanopyri at the time of data gathering. The requirement to find the completeness and contamination of the genome stems from our previous study where all the 139 genomes of Halobacteria were initially considered for pangenome analysis and unfortunately, the core genes counted to zero. Even the soft-core (Fig. 1) that contains clusters in at-least 95% of the genomes was just 207. This was obviously due to incompleteness of the genomes available in NCBI. Finally, all the selected genomes were annotated using Prokka^20^ to find the protein coding genes of each genome. The gene size ranges from 2137 to 4750.

**Table 1:** List of 17 Euryarchaeota genomes as close relatives of Halobacteria with completeness, contamination and the corresponding class.

Organism name	Class	Completeness (%)	Contamination
*Methanothermobacter thermautotrophicus* str. Delta H	Methanobacteria	100	0.359
*Methanobrevibacter smithii* ATCC 35061	Methanobacteria	100	0
*Ferroglobus placidus* DSM 10642	Archaeoglobi	100	0
*Archaeoglobus fulgidus* DSM 4304	Archaeoglobi	100	0
*Thermococcus kodakarensis* KOD1	Thermococci	100	0
*Pyrococcus horikoshii* OT3	Thermococci	100	0
*Methanoculleus marisnigri* JR1	Methanomicrobia	100	0.934
*Methanocella paludicola* SANAE	Methanomicrobia	100	0.934
*Methanococcus maripaludis* S2	Methanococci	100	0
*Methanocaldococcus vulcanius* M7	Methanococci	100	0
*Aciduliprofundum boonei* T469	unclassified Euryarchaeota	100	0
*Aciduliprofundum* sp. MAR08-339	unclassified Euryarchaeota	100	0
*Methanopyrus kandleri* AV19	Methanopyri	99.93	0.934
*Methanomassiliicoccus luminyensis* B10	Thermoplasmata	99.07	0.33
*Candidatus Methanomethylophilus alvus* Mx1201	Thermoplasmata	99.07	0.934
*Candidatus Nanosalinarum* sp. J07AB56	Nanohaloarchaea	78.58	3.73
*Candidatus Nanosalina* sp. J07AB43	Nanohaloarchaea	75.39	4.47

### Halobacterial genomes are highly heterogeneous and have an open pangenome

OMCL^21^ algorithm was applied to protein sequences from all the genomes to get the pan Halobacteria comprising of 49,311 clusters. GET HOMOLOGUES^22^ divided the clusters into four categories viz Core, Softcore, Cloud and Shell. Core clusters are the clusters which have sequences from each genome whereas softcore in 95% of the genomes. Cloud can be defined as the gene clusters which have sequences from very few genomes. Its cut-off is based on most populated cluster which is not in core and its neighbouring clusters. Shell genome includes the clusters present in majority of genomes except the soft-core clusters. Pan Halobacteria with its compartments (Core: 300, Softcore: 998, Cloud:36531, Shell:11784) is described in Fig. 1. The strict core clusters which contain exactly one sequence from each taxa were reported to be 225 and can be considered as true orthologs. Alternatively, all the clusters except the core clusters can be called as variable genome clusters. The pan Euryarchaeota consist of total 60,809 clusters and 45 core clusters (Fig. 1). The large number of variable clusters shows the heterogeneity embedded in the class Halobacateriaand cues for horizontal gene transfer.

**Figure 2:**
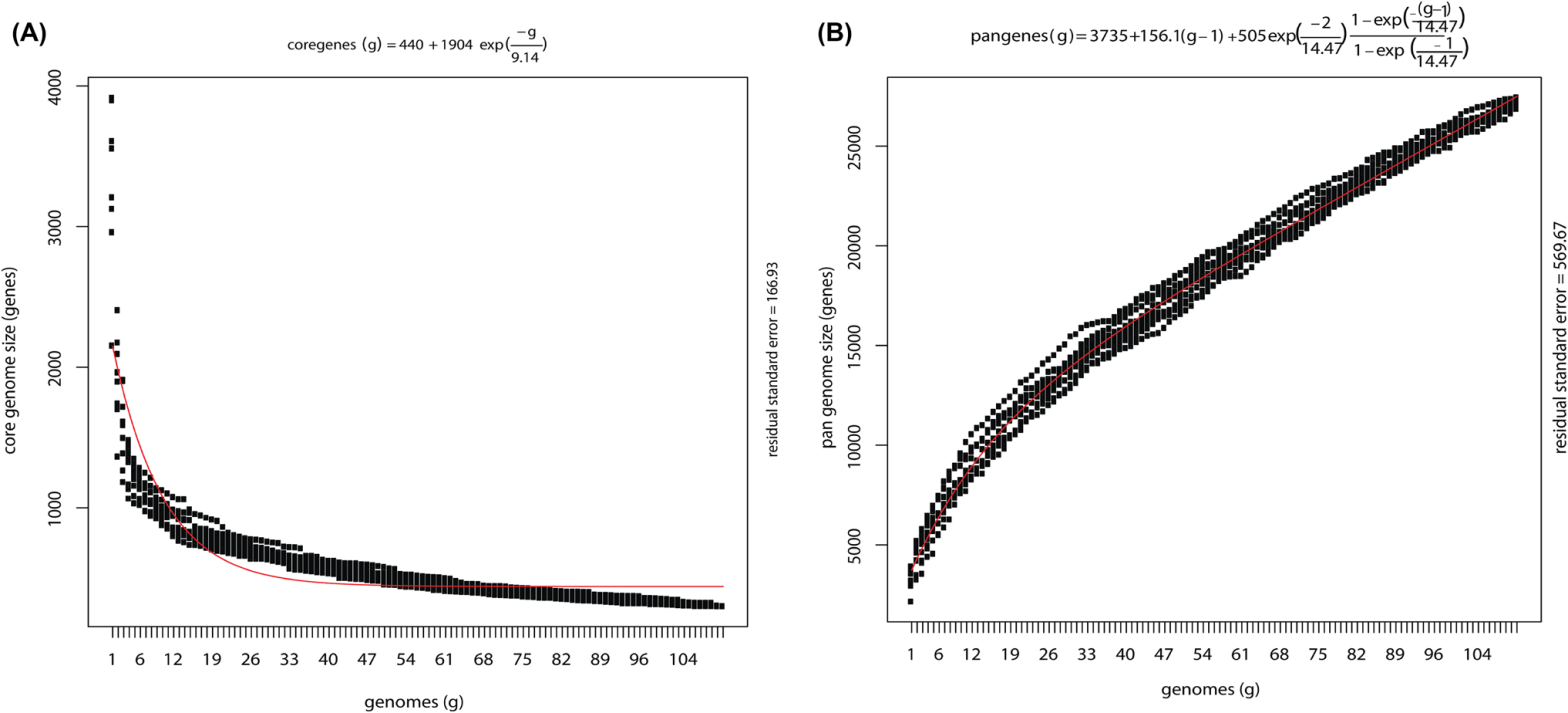
(**A**) Exponential growth curve of pan genes (y-axis) with number of genomes (x-axis) for pan Halobacteria. (**B**) Exponential decay curve of core genes (y-axis) with number of genomes (x-axis) for pan Halobacteria.

The plots of pan genes and core genes against the sampled genomes were also constructed on the basis of exponential model of Tetellin^23^. An exponential growth curve fitting the exponential decay function Fs = κs exp [− n/Țs] + tg(θ) where n is the number of genomes and κs, Țs and tg(θ) are free parameters is shown in Fig. 2A An exponential decay curve is described in Fig. 2B according to the equation Fc = κc exp [− n/Țc] + Ω where κc, Țc and Ω are free parameters and n is the number of genomes. Core gene curve suggests that 71 genomes are enough to find the core of the Halobacteria and as the number of genomes get increased, the pan genes also increases suggesting open pangenome again signalling for horizontal gene transfer among the class. Same trend is shown for the phylum Euryarchaeota (Supplementary Fig. S1).

Genetic material can be horizontally transferred by three processes: conjugation, transformation and transduction. Therefore, to further investigate the role of horizontal gene transfer in evolution of archaea, we downloaded the fasta sequences of the genes responsible for all the three processes using Uniprot KB^24^ with GO^25^ biological process as genetic transfer having these three processes as child terms and blasted the sequences against all the annotated sequences of genomes of Halobacteria. The results showed the presence of genes like DNA protecting protein DprA^26^ and SMF^27^ family protein which can aid in the process of transformation. Other proteins found were conjugal protein and bacterial conjugal protein, members of family "Type IV secretion system protein TraG/VirD4" which play role in conjugation^28^. However, we did not get much information about halobacteria infected by a phage. Nonetheless, the probability of other two mechanisms taking part in horizontal gene transfer can't be underestimated.

### Functional assessment of core gene clusters

**Figure 3:**
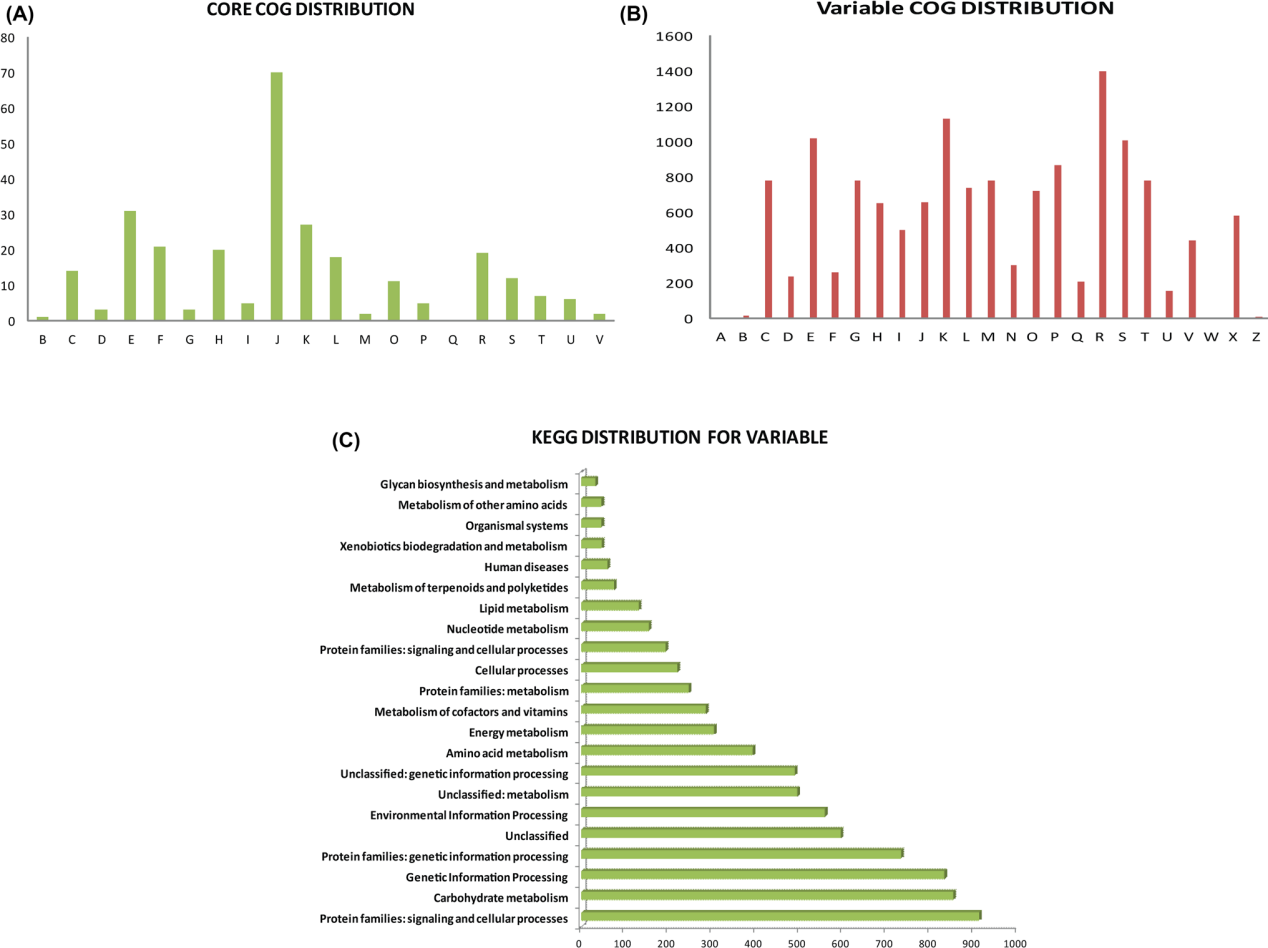
(**A**) Distribution of COG^29^ categories for core clusters and (**B**) variable clusters of pan Halobacteria. (**C**) Distribution of KEGG categories^31^ for variable clusters of pan Halobacteria.

As described in methods section, the represented sequences of core clusters of Halobacteria was annotated using COG (Cluster of Orthologous groups)^29^ resulting in 277 COG annotations out of 300Majority of COG clusters presented in both the Halobacteria core are Translation, ribosomal structure and biogenesis (70), Amino acid metabolism (31), Transcription (27), Nucleotide transport metabolism (21) followed by Coenzyme transport and metabolism (20) and General function prediction (19) (Fig. 3A).

### Membrane transport and signal transduction systems are enriched in the variable parts of halobacterial genomes

Representative sequences of variable clusters showed just 29% and 15.9% annotation against COG and KEGG^30,31^ database respectively. The significant portion as seen in the Fig. 3B represents the COG categories: General function prediction only (1397), Transcription (1129) Signal transduction mechanisms (1036), Amino acid transport and metabolism (1022) followed by Function unknown (1008) and Lipid transport and metabolism (868). In KEGG^31^ categories, the highest being protein families in signal and transduction mechanisms (Fig. 3C). Detailed kegg annotation for genes of variable component are presented in Supplementary Table S4.

### A new superorder comprising halobacteriales and natriabales

**Figure 4:**
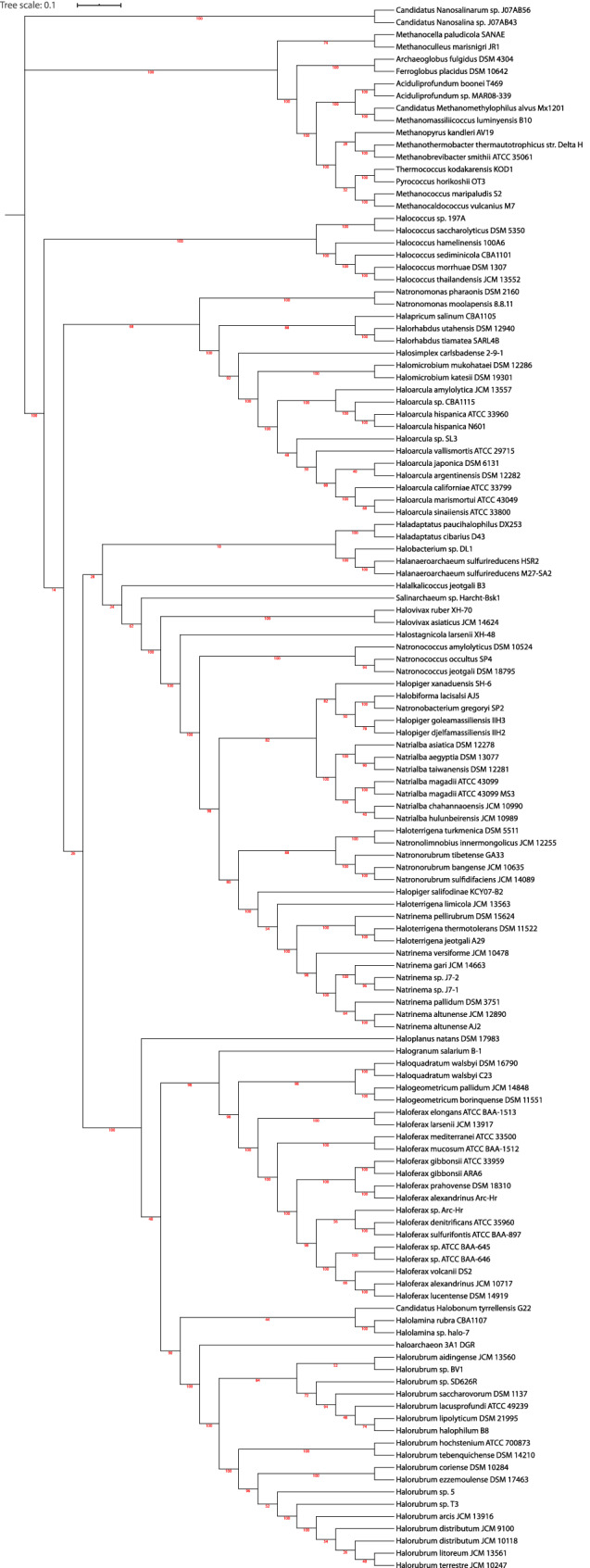
Core gene tree of Euryarchaeota (ME tree).

**Figure 5:**
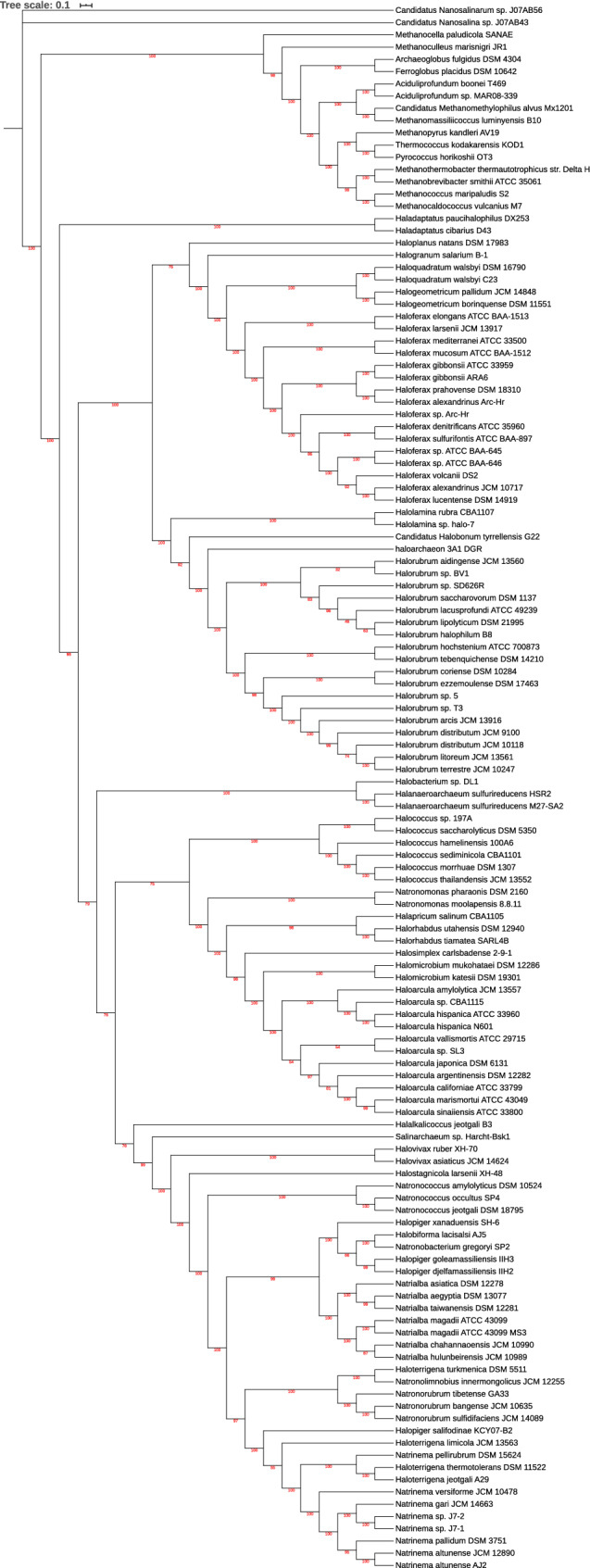
Core gene tree of Euryarchaeota (ML tree).

**Figure 6:**
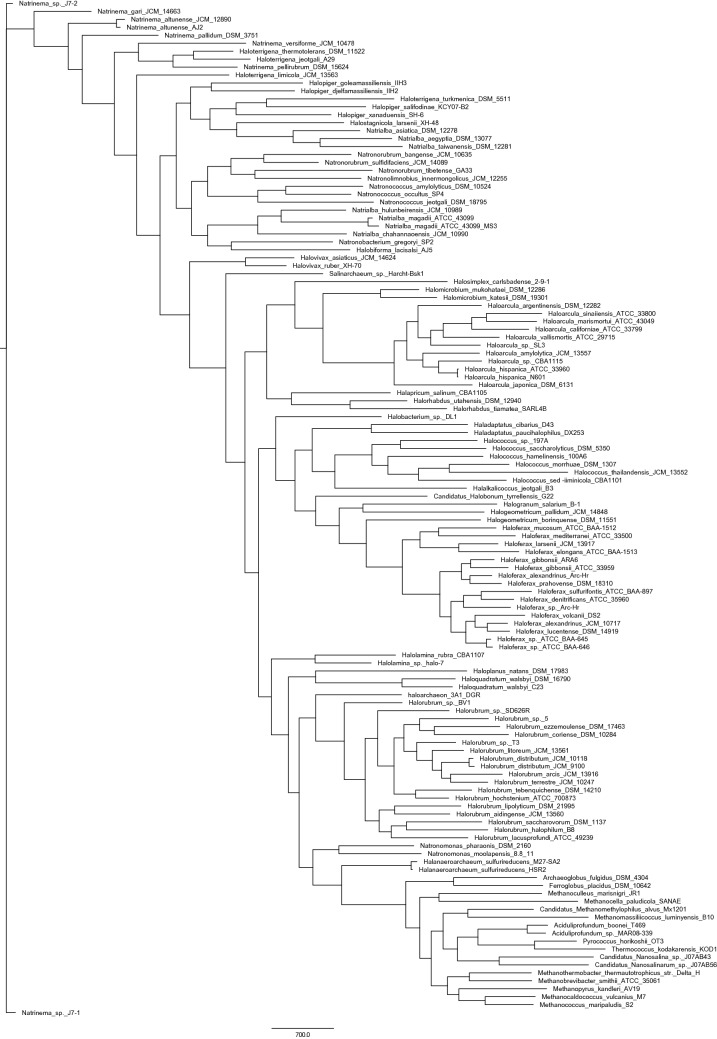
Pan gene tree of Euryarchaeota.

To infer the phylogeny of the class Halobacteria, both the core gene tree of Euryarchaeota (depending on the strict core sequences present in all organisms) and pan gene tree of Euryarchaeota (based on absence and presence of gene families) were constructed. Single gene phylogenies as opposed to multi-gene phylogenies have low discriminatory ability. Thus, multi-gene phylogenies serve a better solution. There have been studies stating the importance of pan-genomic trees in correctly revealing the functional relationship between the organisms. As argued by Snipen, two organisms are similar by presence of same gene families but also by the absence of same gene families^32^. Moreover, the absence of a gene family can give substantial information about the differences in phenotypes among the group of organisms may be as a result of a lost gene in the process of evolution. Usually single gene approach or the housekeeping genes are used to construct phylogenetic tree but these methods do not have high discriminatory power to distinguish between two close species. In Euryarchaeota, specifically in halobacteria, it has been a challenging task to identify a halophilic archaeon, could be due to high dissimilarity of 16sribosimal RNA between halobacteria species. Therefore, a hybrid approach of using both the core genes tree (based on minimum evolution) and pan-gene tree (based on absence and presence of gene families) can accurately find the evolutionary relationship among the members of the class. Both the trees revealed that nanohaloarchea can be treated as an outgroup to haloarchaea. For confirmatory results, maximum likelihood tree with 1000 bootstrap and model "LG + F + R10" found by model finder was also constructed. If not for anything, all the three trees at-least revealed the implication of a new superorder comprising of Halobacteriales and Natrialbales. Core gene tree (minimum evolution), Maximum likelihood tree, Pan gene tree are shown in Figs. 4, 5 and 6 respectively. Single gene trees are also available as supplementary information on zenodo https://doi.org/10.5281/zenodo.4015722.

### Genome expansion during the evolution of halobacteria

**Figure 7:**
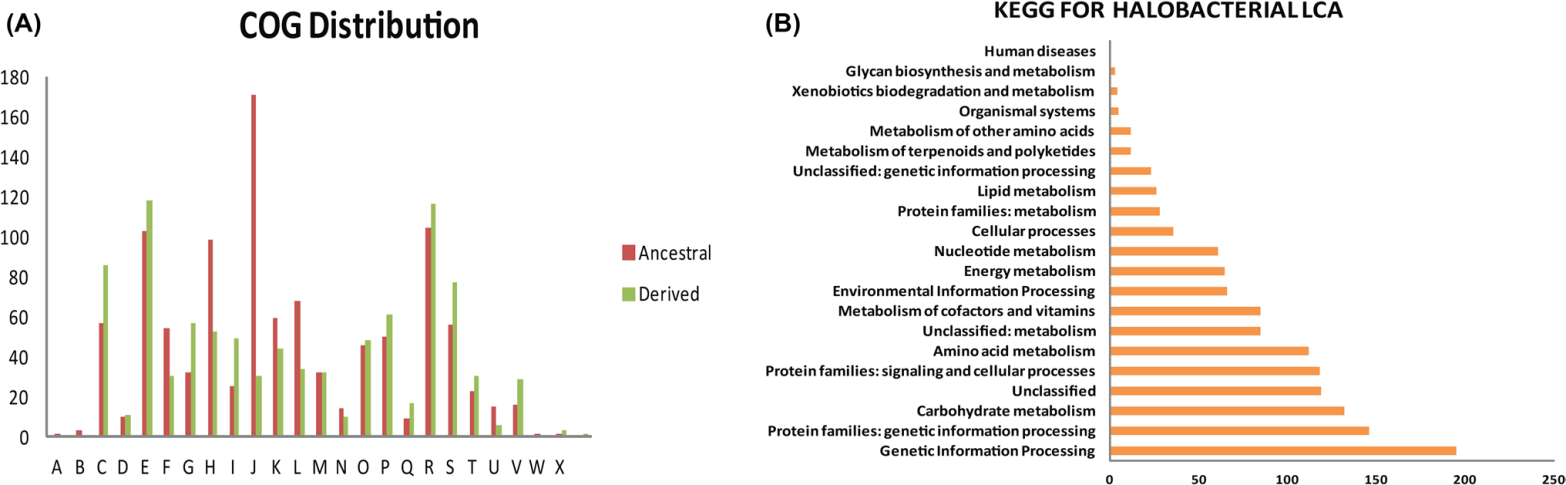
(**A**) Distribution of COG^29^ categories for ancestral and derived genes for halobacterial LCA. (**B**) Distribution of KEGG^31^ categories for total genes of Halobacterial LCA.

Ancestral gene sequences of Halobacterial class was constructed by taking pan matrix of Euryarchaeota so that 17 genomes can be included as close relative of Halobacteria class. This matrix as discussed earlier has rows as gene families and columns represent the number of members in the taxa of that family. The core-gene maximum likelihood tree of the Euryarchaeota can be taken as guide tree onto which the phyletic pattern of presence and absence of gene families can be mapped. The method used here was Wagner parsimony implemented by the java application Count^33^ which resulted in the information of the genes present in the Last Common Ancestor (LCA) of Halobacteria (red oval shaped) in core ML tree. The LCA of class Halobacteria contained 2491 gene families outcome of gain of 1374 gene families and a loss of 32 gene families. Ancestral genome reconstruction^34^ can divide the genes into ancestral sequences and derived sequences. Sequences which are present before the LCA are called as ancestral sequences whereas the sequences which are gained at the LCA node are called ad derived sequences. Thus, in our study, derived sequences accounted to 1374 gene families whereas ancestral sequences comprised of 1117 gene families. The high percentage (55.1%) of derived genes also suggest the presence of HGT^35^ events taking place in the evolution of Halobacteria, as horizontal movement of genes are more rapid than the vertical transfer. Information about various gene gain and loss events at each node and leaf are presented in Supplementary Table S5 where $251_noname is a Last Common Ancestral Node of Halobacteria. Gene count with respect to gene families is presented in Supplementary Table S6. The percentage of ancestral and derived sequences annotated against COG^29^ database were 93.8% and 68.4% respectively (Fig. 7A). The complete pathways in Halobacteria Common Ancestor deduced using KEGG^31^ are shown in Fig. 7B (54.6% annotated) and information on modules are presented in Table 2. Detailed annotation of ancestral and derived genes can be found in Supplementary Table S7 as separate sheets. We also investigated the probable gene transfers from eubacteria in the halobacterial LCA, and we found 858 genes from which 499 are ancestral and 359 derived. Listing of these genes with COG^29^ annotation are provided in Supplementary Table S8.

**Table 2:** Kegg modules for halobacterial last common ancestor.

Pathway modules
**Carbohydrate metabolism**
*Central carbohydrate metabolism*
M00002 Glycolysis, core module involving three-carbon compounds (6) (complete)
M00009 Citrate cycle (TCA cycle, Krebs cycle) (13) (complete)
M00010 Citrate cycle, first carbon oxidation, oxaloacetate ⇒ 2-oxoglutarate (3) (complete)
M00011 Citrate cycle, second carbon oxidation, 2-oxoglutarate ⇒ oxaloacetate (10) (complete)
M00005 PRPP biosynthesis, ribose 5P ⇒ PRPP (1) (complete)
*Other carbohydrate metabolism*
M00012 Glyoxylate cycle (5) (complete)
M00741 Propanoyl-CoA metabolism, propanoyl-CoA ⇒ succinyl-CoA (6) (complete)
**Energy metabolism**
*Carbon fixation*
M00168 CAM (Crassulacean acid metabolism), dark (2) (complete)
*Methane metabolism*
M00378 F420 biosynthesis (5) (complete)
*Nitrogen metabolism*
M00531 Assimilatory nitrate reduction, nitrate ⇒ ammonia (2) (complete)
*ATP synthesis*
M00149 Succinate dehydrogenase, prokaryotes (4) (complete)
M00155 Cytochrome c oxidase, prokaryotes (5) (complete)
M00159 V-type ATPase, prokaryotes (9) (complete)
**Lipid metabolism**
*Fatty acid metabolism*
M00086 beta-Oxidation, acyl-CoA synthesis (1) (complete)
**Nucleotide metabolism**
*Purine metabolism*
M00048 Inosine monophosphate biosynthesis, PRPP + glutamine ⇒ IMP (12) (complete)
M00049 Adenine ribonucleotide biosynthesis, IMP ⇒ ADP,ATP (6) (complete)
*Pyrimidine metabolism*
M00051 Uridine monophosphate biosynthesis, glutamine (+ PRPP) ⇒ UMP (8) (complete)
M00052 Pyrimidine ribonucleotide biosynthesis, UMP ⇒ UDP/UTP,CDP/CTP (3) (complete)
**Amino acid metabolism**
*Serine and threonine metabolism*
M00018 Threonine biosynthesis, aspartate ⇒ homoserine ⇒ threonine (5) (complete)
*Branched-chain amino acid metabolism*
M00019 Valine/isoleucine biosynthesis, pyruvate ⇒ valine / 2-oxobutanoate ⇒ isoleucine (5) (complete)
M00535 Isoleucine biosynthesis, pyruvate ⇒ 2-oxobutanoate (4) (complete)
M00570 Isoleucine biosynthesis, threonine ⇒ 2-oxobutanoate ⇒ isoleucine (6) (complete)
M00432 Leucine biosynthesis, 2-oxoisovalerate ⇒ 2-oxoisocaproate (4) (complete)
*Arginine and proline metabolism*
M00844 Arginine biosynthesis, ornithine ⇒ arginine (3) (complete)
M00015 Proline biosynthesis, glutamate ⇒ proline (3) (complete)
*Histidine metabolism*
M00026 Histidine biosynthesis, PRPP ⇒ histidine (10) (complete)
M00045 Histidine degradation, histidine ⇒ N-formiminoglutamate ⇒ glutamate (4) (complete)
*Aromatic amino acid metabolism*
M00023 Tryptophan biosynthesis, chorismate ⇒ tryptophan (7) (complete)
**Metabolism of cofactors and vitamins**
*Cofactor and vitamin metabolism*
M00880 Molybdenum cofactor biosynthesis, GTP ⇒ molybdenum cofactor (5) (complete)
M00846 Siroheme biosynthesis, glutamate ⇒ siroheme (8) (complete)
**Signature modules**
**Module set**
*Metabolic capacity*
M00615 Nitrate assimilation (1) (complete)

## Discussion

The Halobacteria or more precisely haloarchaea are specialised microorganisms which are capable of surviving in high stress environments including high UV radiation, high salt, nutrient depletion and oxygen stress. Their remarkable feature of longevity has grabbed the interest of scientific community most importantly in agricultural science where scientists strive to produce high resistant crops which can use barren salt lands as well as can survive in high UV environment^2^. Therefore, studying its genomic features and how they got adapted to certain environment would be beneficial for the society. Earlier, the studies for identification of genomic features were expensive and time consuming, but with the advent of next generation sequencing, studying genomes has become quite approachable and reasonable. Number of genomes of halobacterial genomes has vastly increased in last few years. Thus, this has given us opportunity to explore all the halobacterial genomes with the help of pangenome analysis. For this, we collected the genome data of all halobacterial species available in NCBI^36^ at the time of data gathering which counted to 139. However, some genomes are not complete in the dataset and for pangenome analysis, the initial dataset is very important. So, we only selected those halobacterial genomes (111) which were complete (more than 99%) and contamination less than 5%. However, 17 genomes of phylum Eyurarcheota were also taken into our study as the close relatives of Euryarchaeota and to find the correct outgroup for the phylogenetic tree. Complete genomes in accessing horizontal gene transfer, inferring correct phylogenies and ancestral states are indispensable. Our study is one of the few that have not neglected this crucial step.

Any pangenomic study has four important characteristics.

1. A plot of pan genes versus number of genomes.
2. A plot of core gene versus number of genomes.
3. Variable clusters—shell cluster plus cloud clusters..
4. Core clusters- present in all the genome studied.
5. core genome tree and pan genome tree.

Pan and core genome size plots are very essential to any pangenomic study. These are important to get the complete set of pan or core genes. In other words, these show the minimum number of genomes required to get the complete core or pangenome which explains that pangenome size tends to increase with increasing number of genomes and does not reach an asymptote limit. Thus, pan halobacteria is an open pangenome which means with every new genome being added, it brings approximately 137 new genes to the pool of pan-genes. The open pangenome again points out to the great diversity embedded in the class "Halobacteria". This is against the notion of having closed pangenomes in isolated environments and that the open pangenome is a characteristic of taxonomy group which can inhabitant multiple environments and can easily get new genes to their pool. Halophilic archaea seem to have a defined environmental condition of high salinity but still have lot of heterogeneity among them. High percentage of variable clusters specifically the shell clusters and a low number of core clusters suggests that the class "Halobacteria" has diverse genetic information embedded in it pointing again out to its heterogeneity. The open pangenome and the large number of variable clusters implicated on horizontal transfer might play role in evolution of bacteria is in agreement with the earlier research^15–17^. Our investigation for the presence of the genes for the three processes of lateral gene transfer (conjugation, transduction and transformation) confirmed it although not all haloarchaeal genomes poses these genes.

Comparison of individual proteins of both the cores of Halobacteria and Euryarchaeota was done to get the proteins specifically conserved in this class and to examine the genes responsible for not only having salt tolerance properties but also for the factors involved in tolerating oxygen and nutrient depleted environments and thus for its longevity. Unique to core proteins of Halobacteria involved 50S ribosomal protein subunits L24P, L29P, L30P and L31e and 30S ribosomal protein S9. Transcriptional regulator PadR-like family protein^37^ and transcriptional regulator PhoU^38^ involved in negative regulation of phenolic and phosphate metabolism were also conserved. HTH-type transcriptional regulator LysM and HTH-type transcriptional regulator Ptr were also found which are the members of AsnC family^38^. These are the regulators of potassium ion transport probably aiding in maintaining potassium concentration in response to high saline environments. Transcription initiation factor IIB was also found which is thought to be a temperature responsive factor. A bacterial regulatory protein, arsR family was also found to be conserved and definitely plays role in high arsenic or antimony exposure because it negatively regulates the ars operon coding for arsenic reductase and thus regulate the thereby preventing the cell from damage^39^. Transcription initiation factor TF2B^38^ is also present in all the 111 halobacterial species as expected. It initiates the transcription in archaea and some studies have also shown that the activity of TF2B increases with heat shock^40^. The conservation of all these proteins show a high transcriptional mechanism taking place in these specialised archaea.

Many DNA recombination and repair proteins were also reported in the core. DNA mismatch repair protein MutS and DNA repair^41^ and recombination protein RadA^42^, possibly help Halobacteria to repair its DNA damaged by UV radiation thus enhancing its longevity. Moreover, some studies have also shown that DNA can act as a nutrient source for some halophilic archaea. DNA integrity scanning protein DisA is also conserved and is known to check for DNA lesions and repairs DNA specifically at the time of sporulation^43^.

Various stress proteins like Universal stress protein^44^, Cold shock like protein CspC^45^, phage shock protein PspA^46^ which play role in various stress environments are also conserved. PspA for example is related to membrane stress and is known to bind the transcriptional activator PspF and thus forming inhibitor complex PspA-PspF in non-stress conditions^47^. On the onset of any stress condition which disturbs the membrane results in disruption of the inhibitor complex and the PspF is free to active the operon resulting in effector functions to control any membrane damage. Therefore, this Psp system is important for membrane integrity^46^. One more important protein called Winged helix-turn-helix transcription repressor (HrcA DNA-binding)^48^ is also found to be conserved. In bacteria, it is known to negatively regulate the chaperons GroeL and DnaK which gets induced by heat stress^48^.

Ubiquinone/menaquinone biosynthesis methyltransferase, an important gene for the biosynthesis of Ubiquinone and menaquinone was also found. These quinone compounds are membrane bound and are crucial for electron transport^49^. One of the earlier studies also showed core clusters of Halobacteria albeit with less number of genomes and completeness and contamination were not taken into account but we could not find much difference in the functionality of the core^13^ except they found several ABC transporters. In our study, we found only one phosphate transport protein Pit A responsible for inorganic phosphate cation symport, Putative branched-chain amino acid transport ATP-binding protein and Molybdate/tungstate import ATP-binding protein WtpC probably for the metal import. Infact, several ABC transporters were found in the variable component of halobacteria pangenome in our study. ABC transporters are widely distributed in the domian Archaea and are responsible for ATP coupled transport of many substrates across the cell membranes^50^. Another important group enriched in variable clusters were Two Component System (TCS). Two component system as the name suggests consists of one sensor component (histidine kinase) which sense changes in the environment and respond through response regulator. Phylogenetic reports reveal that the two component system has migrated from Bacteria to Archaea and Euryarchaeota by the process of lateral gene transfer^51^. The other significant category is cellular component including the largest portion of genes in quorum sensing. Quorom sensing is a process wherein microorganisms regulate gene expression as a result of high cell density^52^. Kate Montgomery et al. showed the evidence of diketopiperazines in *Haloterrigena hispanica*^53^ interacting with N-acyl homoserine lactone produced by bacteria indicating archaeon's ability to communicate with bacteria in mixed populations. Quorum sensing also controls the process of conjugation and horizontal gene transfer.

Construction of pan and core genome tree was also necessary to infer the correct phylogeny of the class because trees based on 16s ribosomal RNA is not sufficient to get the correct placement of organism. Moreover, there is lot of diversity in 16s ribosomal RNA in many halobacterial species, thus finding a multi-gene approach is essential for phylogenetics of Halobacteria. Also, taxonomic identification of novel haloarcheaon will be easier. Unrooted core tree (Minimum evolution) resulted in the archeaon nanohaloarcheaon to be farthest to class "Halobacteria" Thus, the same outgroup was used to develop rooted maximum likelihood core tree, There have been lot of reports showing the best performance of maximum likelihood method over other methods based on best phylogenetic model inferred by model finder. Pan tree is based on absence and presence of 60,809 gene families in 128 organisms (111 haloarchaea plus 17 other Euryarchaeota). Pan tree is essential as two organisms could be similar by the absence of same set of genes. The implications based on all the three trees are as follows:

There is a clear division of class Halobacteria and other classes in the tree making another clade (red box) which suggests that these may be a part of one common ancestor within the phylum Euryarchaeota. Although, clear indication of *Candidatus Nanosalina* sp. J07AB43^19^ and Candidatus Nanosalina sp. J07AB43^19^ can't be given at this time because of the incompleteness of the two genomes. In pan-gene tree, there is no clear division of classes in the phylum Euryarchaeota. Moreover, all the other seven classes of Euryarchaeota are in one clade (red box) but appear to be closely related to order Halobacteriales^5^.

According to NCBI, *Halalkalicocus jeotgali B3* (yellow) is a member of order Halobacteriales and family Halobacteriaceae but in all the trees, the organism is present in the clade of Natrialbaceae^5^ (blue box).

*Candidatus Halobonum tyrrellensis* G22^54^ (green) based on the naming of NCBI, is a member of order Halobacteriales^5^ but the family is still unidentified. This archeaon is embedded in the family Haloferaceae and family Haloruberaceae in pangenomic and core genomic tree respectively. Thus, in any of the case is true, there is no doubt that this archaeon is a member of order Haloferacales^5^.

Similarly, Haloarchaeon-3A1-DGR^55^ (blue) is also placed in Halobacteriales^5^ in NCBI, but seem to be closer to Halorubra (order: Haloferacales) in all the trees.

Looking at the broader aspect, there is a clear division of order Haloferacales^5^ having two families namely Haloferaceceae^5^ and Halorubraceae^5^ in the class Halobacteria. However, the family Halobacteriaceae^5^ of order Halobacteriales^5^ seem to be closely related to Order Natrialbales^5^ than to its sister family Haloarculaceae^5^ of the same order. In fact, in core genomic trees, there are two clear branches leading one to Haloferacales^5^ and other branch leading to other two orders (Natrialbales and Halobacteriales) with their organisms very closely related to each other. A more research on this ambiguity would give us a clear picture of very close relationship between Natrialbales^5^ and Halobacteriales^5^ or the probability of super-order within the class Halobacteria comprising of natrialbales and Halobacteriales cannot be underestimated.

To understand the evolution of Halobacteria more comprehensively, Ancestral State Reconstruction was also performed which can be defined as a process of extrapolating back to reveal hidden ancestral characters based on present observed characters^56^. It has been used to study the evolution of some bacteria in recent years^34,57^ but no study of this type has been reported in literature on haloarchaea. With the advent of various technologies, it has been now possible to construct the ancestral sequences of group of organisms based on their gene content and similarity among them and to see which gene sequences are ancestral and which has been derived at the last common ancestor of halobacteria. Though previous research has suggested the influx of eubacterial genes onto the haloarcheal LCA, but none of them has studied the complete gene repertoire of the haloarchaeal LCA^17^.

Looking at the average number of genes in present Halobacteria which is around 3200 as opposed to lower 2491 gene families present in halobacterial LCA, it can be established that genome expansion has taken place in the evolution of Halobacteria. Williams et al. also confirmed about last common ancestor of archaea as a small sized genome expanding due to HGTs and duplication^15^. A lot of gene gain events as compared to loss events have occurred at both leaves and nodes of the tree, which might be giving them a new adaptation mechanism to various stresses (Supplementary Table S5). Therefore, a need was felt to annotate these derived sequences which were gained at the LCA node. On mapping the gene pool of haloarcheal common ancestor to pathway modules of KEGG database, we found complete modules of (M00002) Glycolysis, core module involving three-carbon compounds, (M00009) Citrate cycle (TCA cycle, Krebs cycle), (M00010) Citrate cycle, first carbon oxidation, oxaloacetate ⇒ 2-oxoglutarate, (M00011) Citrate cycle, second carbon oxidation, 2-oxoglutarate ⇒ oxaloacetate, (M00005). Three modules of energy metabolism namely (M00149) Succinate dehydrogenase, Cytochrome c oxidase, and M00159 V-type ATPase gives clear indication of that the last common ancestor of haloarchaea was able to invest in oxidative phosphyralation might be due to oxidizing environment during that time as suggested by William et al. Other modules worth mentioning are (M00378) F420 biosynthesis in methane metabolism, and (M00531) Assimilatory nitrate reduction, nitrate ⇒ ammonia in nitrogen metabolism. Detailed modules are presented in Table 2.

We also divided the gene pool of the last common ancestor into ancestral (1117) and derived sequences (1374) and as expected, mapping ancestral genes to KEGG database^31^, reveals genes for biosynthesis of F420 (methane metabolism). Thus, supporting the hypothesis of going back to Euryarchaeota common ancestor as methanogenic^15,17^. Many important unique derived genes ie genes that are not already present in ancestral sequences and acquired at the root are found such as subunits for cytochorme c oxidades, NADH quinone oxidoreductases, ferrodoxin, succinate dehydrogenase/fumarate reductase, cytochrome b subunit, iron-sulfur subunit and membrane anchor subunit giving them ability to phosphoralyse oxidatively. Moreover, many genes for glycolysis and TCA cycle like 2,3-bisphosphoglycerate-independent phosphoglycerate mutase, citrate synthase, aconitate hydratase, phosphoenolpyruvate carboxykinase and pyruvate ferredoxin oxidoreductase beta subunit were also added at the node in agreement with the previous research^17^. We also found genes for complete module of nitrogen metabolism in derived genes revealing that these genes were also acquired at halobacterial common ancestor node giving rise to modern day halophilic archaea involving nitrogen metabolism. Though previous research has shown many metabolic genes acquired by the HLCA^17^ but importance of Dna repair proteins and chaperons cannot be underestimated as halophilic archeae are known to reside in extreme environments. Examples of mismatch repair derived proteins are mismatch repair protein MutL, DNA repair protein SbcD/Mre11, DNA-3-methyladenine glycosylase II and DNA ligase (NAD +). Chaperone serine proteases and monothiol glutaredoxin were also acquired. A very recent research on serine protease in helicobacter pyori has claimed to function under stress conditions^58^. Membrane trafficking genes such as ArsR family transcriptional regulator, arsenate/arsenite/antimonite-responsive transcriptional repressor and CU + /H + antiporter were also acquired giving them resistance to high metal toxicity. Many ABC transporters and genes for Quorum sensing were also found**.** ABC proteins play role in influx and efflux of metals in the cell and thus responsible for metal homeostasis^50^ . Two component systems consist of Histidine Kinase sensor which is auto-phosphorylated upon sensing an external stimulus and transfers the phosphate group to Response regulator which in turn is connected to many effecter domains^51^. Though TCS are common in bacteria but less identified in archaea and has not been characterized in halobacteria. Recently, it has been characterised in *Methanosaeta harundinace*^59^. Major portion of proteins also fall into Unclassified category. These are the ones whose relation to any molecular network is unknown. This gives the scientific community an open area of research where gene repertoire of halobacterial ancestor can be investigated their role in pathways can be deduced.

## Methodology

### Genome dataset

A total of 615 archaea genomes were present at the time of data acquisition (Nov, 2015), out of which 430 genomes belonged to phylum Euryarchaeota were downloaded from NCBI after removing the partial assemblies. According to NCBI^36^, a partial assembly is the one where a part of genome is selected. A halobacterial class consisted of non-redundant 139 genomes extending across 36 genera isolated from different regions of the world comprising of both complete genomes and draft assemblies.

### Estimating completeness and annotation

Thus, a strategy of checkM^60^ was applied to estimate the percentage completeness of the genomes. The CheckM^60^ tool was used to find the completeness of the available genomes in the phylum Euryarchaeota. CheckM^60^ employs marker sets which are a group of consistently co-located marker genes to estimate the completeness of the genome. Here, CheckM with archaeal specific marker sets were applied because it uses less computation time and the absolute error of the output was less than 1%. Only, the genomes greater than 99% completeness were chosen for further analysis. Since the number of genomes for Euryarchaeota is too large, only two genomes of each class viz Methanobacteria, Archaeoglobi, Thermococci, Methanomicrobia, Methanococci, Methanopyri, Thermoplasmata, Nanohaloarchaea^19^ and Aciduliprofundum^61^ except Halobacteria were shortlisted which can be considered as close relatives of halobacterial species. However, all genomes of class Halobacteria having greater than 99% completeness were selected for the analysis.

The coding sequences for each genome was predicted using Prokka^20^ based on an efficient algorithm of Prodigal which is known to reduce false positives compared to other gene finding tools^20^. Prokka was run as a command line using default parameters and resulted in CDS translation files in .faa format.

### Clustering

GET HOMOLOOGUES tool^22^ was used to cluster the annotated protein sequences of all the selected genomes on the basis of completeness of Halobacteria and Euryarchaeota. GET HOMOLOGUES applies three strategies for clustering namely BDBH (biderectional best hit), COG^29^ (Cluster of orthologous sequences) and OMCL^21^ (Ortho Markov clustering). OMCL^21^ as the name suggests uses markov clustering algorithm to produce orthologs and paralogs. In our case, OMCL was used because it is highly sensitive in finding orthologous. Minimum coverage of 75% of pair-wise alignment and a cut-off e value of 1e-10 were imposed and resulted in cluster files.

### Estimating the pan-genome

The auxiliary script, compare clusters.pl given by GET HOMOLOGUES^22^ was used to form the pan-genome matrix in text and phylip format from the cluster files of the three datasets. The core, softcore, shell and cloud genes were estimated using the script parse pangenome matrix.pl. The paralog clusters were defined as the clusters having more than one copy from the single genome. These paralog clusters were removed from core clusters using the custom made perl script to get the strict core clusters which contains true orthologs.

### Construction of core and pan-genomic tree

The pangenomic tree was constructed from the pangenome matrix of pan Euryarchaeota in phylip format using the PARS program of the Phylip suite of tools^62^ which works on unordered multistate parsimony^63^. Parsimony algorithm can work on matrix of discrete data having 0 and 1 as absence and presence of gene family respectively. Core tree construction was done using the strict core clusters of Euryarchaeota. Individual clusters were subjected to multiple sequence alignment by muscle with maximum iteration as 8^64^ and the alignments were concatenated by catfastatophylip.pl script. The concatenated alignment was then used to construct the core genome tree using the minimum evolution method^65^ of Mega7 suite^66^ with a bootstrap value of 500^67^. Poison distribution method was used for building the distance matrix^68^. The initial tree was built using Neighbour joining method^69^ whereas Close-Neighbor-Interchange algorithm (CNI)^70^ was used to search the ME tree. Model finder was employed to find the best evolutionary model and the maximum likelihood tree was produced using iqtree with SH-aLRT test and ultrabootstrap value and of 1000. A reliable clade would have SH-aLRT and UFbootstrap value ≥ 85% and ≥ 95% respectively. Figtree 1.4.3 was used for the visualization of the tree.

### Functional classification of strict core and pan genes

The representative genes of the core clusters and the non-core variable clusters forbpan Halobacteria were classified into COG^29^ sscategories using CD (Conserved Domain)^71,½72^ search and BLAST-KOALA^30^ was done for variable component only.CD-BATCH^72^ uses RPS-BLAST (Reverse-PSI) to output pair-wise alignment between query protein sequence and subject domain sequence whereas BLAST KOALA^30^ uses BLASTP against the non-redundant GENES Database and K number assignment is done using weighted sum on bit scores. A default parameter of E-value of 0.01 and filter for low complexity regions against the COG database were used to reduce false positive results for CD search algorithm.

### Estimating core and pan-genome size

Core and pangenome size were calculated as a function of number of genomes for both the halobacterial and Euryarchaeota datasets. Thus genomes were sampled to get the total number of core and pan genes every time the new genome is being added to the dataset. The exponential decay and growth curves were plotted for core and pangenome respectively using plot pancore matrix.pl script given by GET HOMOLOGUES^22^ with the Tettelin parameters^23^.

### Ancestral state reconstruction

The ancestral state was reconstructed using Wagner Parsimony^73^ by setting gene gain/loss cost to 2^74^. Gene gain and loss analysis were performed using Count^33^ based on the pangenome matrix of pan Euryarchaeota and the core gene ML tree acted as a guided tree for the analysis. The representative sequences of Halobacterial LCA, its ancestral and derived clusters were annotated against KEGG database using Blastkoala^30^ Reverse bidirectional blast was performed among the gene pool of the LCA and nr database with applying a filter of taxid 2 (eubacteria) using command line and top hits with minimum 30 percent identity and e value < 1 * 10^–10^ were retained.

## Conclusions

We have performed pangenome analysis of complete genomes of halobacterial class revealing an open pangenome of the class. Across 111 halobacterial species, the core genes corresponded to various transcriptional regulators like PadR-like family protein^37^, PhoU^38^, HTH-type transcriptional regulator LysM^38^, HTH-type transcriptional regulator Ptr^39^, Transcription initiation factor TF2B^38^. Repair genes such as DNA mismatch repair protein MutS^41^ and DNA repair and recombination protein RadA^42^ were also found in accordance with previous research. Cold shock protein^45^, Universal stress protein and phage shock protein^46^ were also found to be conserved in the halobacterial lineage. Though earlier research has focussed on identifying the core genome of Halobacteria, the total genetic variability can only be studied through dispensable part of the genome which was not focussed earlier. The dispensable part of the genome included genes of Quorum sensing^52^ and Two component system^59^ playing role in adapting to different environments. There were many hypothetical proteins in dispensable part of the genome revealing that there are genes whose function is still unknown. The phylogenetic analysis showed that the family Halobacteriaceae^5^ of order Halobacteriales^5^ seem to be closely related to Order Natrialbales^5^ than to its sister family Haloarculaceae^5^ of the same order. The ancestral state reconstruction was also performed which gave information about the Last Common Ancestor of Halobacteria which had gained 1085 genes and had 1371 ancestral genes indicating genome expansion of halobacteria. The ancestral genes were studied and found to be genes of basic processes like replication, transcription and translation whereas the derived genes were more of environmental genes which probably have given them adaptability when migrated to different environments. Our study also showed that horizontal gene transfer (HGT) is the mechanism of transfer of genes between halobacterial species.

## Supplementary information

Supplementary Information 1.

Supplementary Information 2.

Supplementary Information 3.

Supplementary Information 4.

Supplementary Information 5.
