# Supplementary material for: Pan-genome analysis and ancestral state reconstruction of class halobacteria: probability of a new super-order
Source: Sci Rep. 2020 Dec 3;10:21205. doi: 10.1038/s41598-020-77723-6 (PMC7713125; doi:10.1038/s41598-020-77723-6)
Supplement: Supplementary file 1 — Supplementary Information 1. [file 41598_2020_77723_MOESM1_ESM.pdf]

Title: Pan-Genome Analysis and Ancestral State Reconstruction of class Halobacteria: Probability of a new Super-order

Sonam Gaba[1], Abha Kumari[2], Marnix Medema[3] , *Rajeev Kaushik

[1]Division of Microbiology, ICAR-Indian Agricultural Research Institute, New Delhi, INDIA

[2]Amity Institute of Biotechnology, Amity University, NOIDA, Uttar Pradesh, INDIA

[3]Bioinformatics group, Wageningen University, Netherlands

*Corresponding Author, Principal Scientist, Division of Microbiology, ICAR-Indian Agricultural Research Institute, New Delhi, INDIA

Correspondence to rajeev_micro@iari.res.in

Supplementary Table S1 List of 139 Halobacterial genomes

Organism Name	Genome Size	GC content	Completeness(%)	Predicted Genes	Contamination
Candidatus_Halobonum_tyrrellensis_G22	3675087	0.70117959	100	3525	0.9346
Haladaptatus_cibarius_D43_HAPD43	3926724	0.57755905	100	4125	0.9346
Haladaptatus_paucihalophilus_DX253	4284805	0.61815158	100	4364	0.9346
Haladaptatus_paucihalophilus_DX253_B208DRAFT	4317540	0.61766378	100	4391	0.9346
Halalkalicoccus_jeotgali_B3	3637498	0.62631712	100	3860	0.9346
Halanaeroarchaeum_sulfurireducens_strain_HSR2	2085482	0.63290884	100	2137	0
Halanaeroarchaeum_sulfurireducens_strain_M27-SA2	2253500	0.62760817	100	2307	0
Halapricum_salinum	3451492	0.63731424	100	3450	0
Haloarcula_californiae_ATCC_33799	4404932	0.60822119	100	4573	0
Haloarcula_hispanica_ATCC_33960	3484189	0.62753685	100	3535	0
Haloarcula_hispanica_N601	3369916	0.62997357	100	3423	0
Haloarcula_japonica_DSM_6131	4280359	0.61176883	100	4384	0.9346
Haloarcula_marismortui_ATCC_43049	4274642	0.61118802	100	4453	0
Haloarcula_sinaiiensis_ATCC_33800	4405164	0.60770723	100	4543	0
Haloarcula_sp._CBA1115	3423144	0.62969948	100	3480	0
Haloarcula_sp._SL3	3970989	0.62048725	100	4056	0
Haloarcula_vallismortis_ATCC_29715	3923205	0.61797586	100	4060	0
Halobiforma_lacisalsi_AJ5	4338576	0.65330565	100	4264	0
Halococcus_hamelinensis_100A6	3419074	0.65398259	100	3516	0.0719
Halococcus_sediminicola	3764367	0.62293315	100	3907	0.9346
Halococcus_thailandensis_JCM_13552	4052434	0.61822054	100	4366	1.8692
Haloferax_alexandrinus_Arc-Hr	3893626	0.66004634	100	3848	0
Haloferax_alexandrinus_JCM_10717	3649262	0.66301141	100	3666	0
Haloferax_denitrificans_ATCC_35960	3825973	0.66286772	100	3809	0
Haloferax_elongans_ATCC_BAA-1513	3952136	0.61154485	100	3994	0
Haloferax_gibbonsii_ATCC_33959	4057322	0.65854867	100	4022	0
Haloferax_gibbonsii_strain_ARA6	2945391	0.67061691	100	3034	0
Haloferax_larsenii_JCM_13917	3697948	0.62036385	100	3714	0
Haloferax_lucentense_DSM_14919	3619064	0.66390173	100	3654	0
Haloferax_prahovense_DSM_18310	3998799	0.65689779	100	3991	0
Haloferax_sp._Arc-Hr	4015175	0.66340729	100	3919	0
Haloferax_sp._ATCC_BAA-645	3598205	0.66645665	100	3616	0
Haloferax_sulfurifontis_ATCC_BAA-897	3812428	0.66301134	100	3850	0
Haloferax_volcanii_DS2	3866754	0.6576704	100	3931	0
Halogeometricum_borinquense_DSM_11551	2820544	0.61063646	100	2905	0
Halolamina_rubra	2955001	0.69048031	100	3123	1.2461
Halolamina_sp._halo-7	2835858	0.67943035	100	2886	0
Halomicrobium_katesii_DSM_19301	3607771	0.65031887	100	3661	0.9346
Halomicrobium_mukohataei_DSM_12286	3110487	0.6562908	100	3198	0
Halopiger_sp._IIH2	3776554	0.64309473	100	3719	0
Halopiger_sp._IIH3	3906364	0.66064335	100	3853	1.2221
Halopiger_xanaduensis_SH-6	3668009	0.6598179	100	3601	0
Halorhabdus_tiamatea_SARL4B	2815791	0.63368553	100	2835	0
Halorhabdus_utahensis_DSM_12940	3116795	0.62899966	100	3043	0.9346
Halorubrum_aidingense_JCM_13560	3108525	0.67190581	100	3062	0.9346

Halorubrum_arcis_JCM_13916	3382601	0.67336675	100	3376	0
Halorubrum_coriense_DSM_10284	3645313	0.6699307	100	3623	0.9346
Halorubrum_distributum_JCM_10118	3306135	0.68082129	100	3223	0
Halorubrum_distributum_JCM_9100	3307369	0.68075036	100	3231	0
Halorubrum_ezzemoulense_DSM_17463	3591480	0.66603127	100	3555	0.4673
Halorubrum_halophilum	3677984	0.65138986	100	3670	0.9346
Halorubrum_hochstenium_ATCC_700873	3037532	0.69130537	100	3008	0
Halorubrum_lacusprofundi_ATCC_49239	3261238	0.65177028	100	3235	0
Halorubrum_lipolyticum_DSM_21995	3425042	0.68012864	100	3308	0
Halorubrum_litoreum_JCM_13561	3137757	0.68903782	100	3100	0
Halorubrum_saccharovorum_DSM_1137	3423703	0.66885064	100	3353	0.9346
Halorubrum_sp._5	3567018	0.67646374	100	3595	0
Halorubrum_sp._BV1	2767463	0.66009743	100	2765	0
Halorubrum_sp._SD626R	3248714	0.67560491	100	3284	3.8821
Halorubrum_sp._T3	3168011	0.68478174	100	3054	0
Halorubrum_tebenquichense_DSM_14210	3328860	0.68033548	100	3354	0
Halorubrum_terrestre_JCM_10247	3376225	0.68029558	100	3361	0
Halosimplex_carlsbadense_2-9-1	4694889	0.67674766	100	4581	1.8692
Halostagnicola_larsenii_XH-48	2789326	0.61947223	100	2871	0
Haloterrigena_jeotgali_A29_HTGA29	4131621	0.64918746	100	4262	0
Haloterrigena_limicola_JCM_13563	3522035	0.61795984	100	3657	1.8692
Haloterrigena_turkmenica_DSM_5511	3889038	0.6583335	100	3753	0
Halovivax_asiaticus_JCM_14624	3238452	0.64454407	100	3215	0
Halovivax_ruber_XH-70	3223876	0.64336035	100	3132	0
Natrialba_asiatica_DSM_12278	4404175	0.62388955	100	4319	0
Natrialba_chahannaoensis_JCM_10990	4309274	0.60417566	100	4262	0
Natrialba_magadii_ATCC_43099	3751858	0.61420821	100	3622	0
Natrinema_altunense_JCM_12890	3774970	0.64549215	100	3814	0
Natrinema_altunense_strain_AJ2_N_altunense_AJ2	3774135	0.64558316	100	3794	0
Natrinema_gari_JCM_14663_	4023692	0.6372259	100	4149	0
Natrinema_pellirubrum_DSM_15624	4264455	0.64124623	100	4336	0
Natrinema_sp._J7-1	3667624	0.64375492	100	3732	0
Natrinema_versiforme_JCM_10478	4190799	0.63956893	100	4285	0
Natronococcus_amylolyticus_DSM_10524	4416525	0.64362843	100	4459	0.9346
Natronococcus_occultus_SP4	4013216	0.64935802	100	4007	0
Natronolimnobius_innermongolicus_JCM_12255	4588634	0.64290534	100	4467	0
Natronomonas_moolapensis_8.8.11	2912573	0.64525833	100	2923	0
Natronomonas_pharaonis_DSM_2160	2595221	0.63440223	100	2679	0
Natronorubrum_bangense_JCM_10635	4111275	0.60391159	100	4167	0.9346
Natronorubrum_sulfidifaciens_JCM_14089	3460288	0.6177978	100	3516	0
Natronorubrum_tibetense_GA33	4934841	0.6225404	100	4750	0.0719
Salinarchaeum_sp._Harcht-Bsk1	3255260	0.66585987	100	3105	0
Halobacterium_sp._DL1	2846968	0.67484988	99.76636	2988	0
haloarchaeon_3A1_DGR	2880902	0.67680959	99.53271	2831	1.8692
Haloarcula_amylolytica_JCM_13557	4225424	0.62127245	99.53271	4327	0.9346
Haloarcula_argentinensis_DSM_12282	4147107	0.61116133	99.53271	4272	0
Haloferax_mediterranei_ATCC_33500	2946877	0.61131681	99.53271	3076	0
Natrialba_aegyptia_DSM_13077	4618362	0.6198454	99.53271	4586	0
Natrialba_hulunbeirensis_JCM_10989	4159606	0.6172775	99.53271	3984	0.4673
Natrialba_taiwanensis_DSM_12281	4635192	0.61521218	99.53271	4604	0

Natronococcus_jeotgali_DSM_18795	4496185	0.64411113	99.53271	4632	0.9346
Natrialba_magadii_ATCC_43099_strain_MS-3	4416299	0.61054588	99.22118	4362	0.9346
Halococcus_agarilyticus	3476728	0.66023706	99.06542	3475	0
Halococcus_morrhuae_DSM_1307	2991556	0.63844997	99.06542	3175	0
Halococcus_saccharolyticus_DSM_5350	3449700	0.64028588	99.06542	3582	1.8692
Haloferax_mucosum_ATCC_BAA-1512	3368982	0.61847704	99.06542	3454	0
Halogeometricum_pallidum_JCM_14848	4384515	0.65645737	99.06542	4349	0.9346
Halogranum_salarium_B-1_162.HSB1.1_1	4492306	0.6220066	99.06542	4528	0
Halopiger_salifodinae_strain_KCY07-B2	4350898	0.65413665	99.06542	4248	3.7383
Haloplanus_natans_DSM_17983	3797935	0.6500759	99.06542	3968	0
Haloquadratum_walsbyi_C23	3148033	0.47782599	99.06542	2984	0
Haloquadratum_walsbyi_DSM_16790	3132494	0.47863907	99.06542	3015	0
Haloterrigena_thermotolerans_DSM_11522	3895275	0.65388889	99.06542	3938	0
Natrinema_pallidum_DSM_3751	3915814	0.63719628	99.06542	3969	0.9346
Natrinema_sp._J7-2	3697626	0.64249023	99.06542	3725	0
Natronobacterium_gregoryi_SP2	3694030	0.62299939	99.06542	3746	0
Haloferax_sp._BAB2207_V.1	3744707	0.65792503	98.99353	4415	0.9346
Halococcus_salifodinae_DSM_8989	4199784	0.63445778	98.59813	4323	0.9346
Halolamina_pelagica_strain_CDK2	2972542	0.67603923	98.59813	3506	0.9346
Haloterrigena_salina_JCM_13891	4841607	0.65169498	98.59813	4652	0
Haloferax_sp._ATCC_BAA-644	3587033	0.66664343	98.13084	3602	0
Halarchaeum_acidiphilum_MH1-52-1	2628974	0.6738374	97.35202	2856	0
Halobacterium_salinarum_R1	2000962	0.68014835	97.19626	2102	0
Halobacterium_sp._NRC-1	2014239	0.67913043	97.19626	2106	0
Halobellus_rufus	3852219	0.64118084	97.19626	3971	0
Halorubrum_kocurii_JCM_14978	3619738	0.66862158	97.19626	3571	0
Halonotius_sp._J07HN6	2529000	0.62639432	96.41745	2837	3.7383
Halobiforma_nitratireducens_JCM_10879	3688747	0.63724616	95.3271	3676	0.9346
Halonotius_sp._J07HN4	2888659	0.60962115	94.15888	3196	24.299
Halostagnicola_sp._A56	3178490	0.60777895	93.54182	3401	1.0065
Halorubrum_saccharovorum_strain_H3_NODE_1	3282373	0.66483364	93.38605	3823	0.9346
Haloquadratum_walsbyi_J07HQW2	3594539	0.47984027	92.13995	3845	1.8692
halophilic_archaeon_J07HX64	2982938	0.64061338	87.86844	3041	2.3364
Natronolimnobius_baerhuensis_JCM_12253	3893062	0.60142479	85.90343	5302	0
Natrinema_altunense_strain_1A4-DGR	3718305	0.64801623	85.83753	5159	0
Haloquadratum_walsbyi_J07HQW1	3475501	0.49416339	84.81908	3534	0
Haloarcula_salaria_strain_H5-DGR	4091500	0.62066259	82.87803	6042	0
halophilic_archaeon_J07HB67	2649547	0.67164904	69.70405	3134	0
Haloquadratum_sp._J07HQX50	3019909	0.50682333	67.3017	3908	0
Halorubrum_sp._AJ67_WGS	4225006	0.63707054	58.17757	4618	0.4673
Halorubrum_sp._J07HR59	2120805	0.59566555	58.09969	3039	0
halophilic_archaeon_J07HX5	2040945	0.60697891	38.78505	2570	2.8037
Haloferax_sp._ATB1	4223705	0.61793886	28.97196	4381	5.6075
Haloterrigena_sp._H13	596279	0.64984345	9.261922	1397	0

Supplementary Table S2 List of 408 total Euryaechaeota genomes

Organism Name	Genome Size	GC content	Completeness (%)	Predicted Genes	Contamination
Acidiplasma aeolicum strain=V	1746790	0.34	97.66	1902	1.87
Acidiplasma aeolicum strain=VT	1778901	0.34	98.13	1947	3.74
Acidiplasma cupricumulans JCM 13668 strain=JCM 13668	1747526	0.34	81	3350	1.87
Acidiplasma cupricumulans strain=BH2	1731076	0.34	98.6	1835	1.87
Acidiplasma sp. MBA-1 strain=MBA-1	1747364	0.34	98.6	1825	1.87
Aciduliprofundum boonei T469 strain=T469	2981805	0.39	92.06	3181	0
Aciduliprofundum boonei T469 strain=T469	1486778	0.39	100	1558	0
Aciduliprofundum sp. MAR08-339 strain=MAR08-339	1437090	0.44	100	1534	0
Archaeoglobus fulgidus DSM 4304 strain=DSM 4304	2178400	0.49	100	2518	0
Archaeoglobus fulgidus DSM 8774 strain=DSM 8774	2316287	0.48	100	2657	0
Archaeoglobus profundus DSM 5631 strain=DSM 5631	1560622	0.42	98.13	1868	0
Archaeoglobus sulfaticallidus PM70-1 strain=PM70-1	2076931	0.43	100	2245	0
Archaeoglobus veneficus SNP6 strain=SNP6	1901943	0.47	100	2153	0.93
Candidatus Methanomassiliicoccus intestinalis Issoire	1931651	0.41	98.6	1855	0.93
Candidatus Methanomethylophilus alvus Mx1201 strain	1666795	0.56	99.07	1636	0.93
Candidatus Methanoperedens nitroreducens ANME-2d	3203386	0.43	99.07	3422	0
Candidatus Methanoperedens sp. DS-2015	3738466	0.4	97.2	4659	6.56
Candidatus Methanoplasma termitum strain=MpT1	1488669	0.49	98.13	1419	1.61
Euryarchaeota archaeon SCGC AAA252-I15 strain	1325681	0.45	62.31	1507	0.93
Euryarchaeota archaeon SCGC AAA286-E23 strain	512945	0.27	15.89	593	0
euryarchaeote SCGC AAA261-E04 strain	758640	0.37	36.16	914	1.94
euryarchaeote SCGC AAA261-G15 strain	965738	0.39	36.41	1192	6.54
Ferroglobus placidus DSM 10642 strain=DSM 10642	2196266	0.44	100	2599	0
Ferroplasma acidarmanus fer1 strain=fer1	1935211	0.36	98.29	1951	0
Ferroplasma sp. Type II	2487652	0.37	95.02	2852	20.1
Geoglobus acetivorans strain=SBH6	1860815	0.47	99.07	2232	0
Geoglobus ahangari strain=234	1770093	0.53	100	2033	0
Marine Group II euryarchaeote SCGC AB-629-J06 strain	534847	0.34	3.666	649	0.5
Methanobacterium arcticum strain=M2	3393923	0.33	99.07	3318	3.74
Methanobacterium formicicum	2478074	0.41	99.07	2410	0
Methanobacterium formicicum DSM 3637 strain	2684267	0.38	98.13	2536	0
Methanobacterium formicicum JCM 10132 strain	2451734	0.41	91.28	3826	0
Methanobacterium formicicum strain=BRM9	2449987	0.41	100	2403	0
Methanobacterium lacus strain=AL-21	2583753	0.36	100	2522	0
Methanobacterium paludis strain=SWAN1	2546541	0.36	99.69	2451	1.87
Methanobacterium sp. Maddingley MBC34	2420154	0.39	96.26	2414	0.07
Methanobacterium sp. MB1	2029766	0.4	97.2	2022	0
Methanobacterium sp. SMA-27 strain=SMA-27	2490725	0.33	99.07	2622	0.93
Methanobacterium veterum strain=MK4	3369555	0.33	99.07	3287	0.93
Methanobrevibacter arboriphilus ANOR1 strain=ANOR1	2221920	0.26	100	2004	0
Methanobrevibacter arboriphilus JCM 13429	2331485	0.25	87.71	3807	0
Methanobrevibacter arboriphilus JCM 9315	2279829	0.26	96.96	2766	0.93

Methanobrevibacter boviskoreani JH1 strain=JH1	2045801	0.29	100	1759	0
Methanobrevibacter oralis JMR01 strain=JMR01	2107831	0.28	95.33	2281	0
Methanobrevibacter ruminantium M1 strain=M1	2937203	0.33	99.93	2199	1.87
Methanobrevibacter smithii ATCC 35061	1853160	0.31	100	1782	0
Methanobrevibacter smithii CAG:186	1729973	0.31	100	1714	0
Methanobrevibacter smithii DSM 2374 strain	1729275	0.31	100	1670	0
Methanobrevibacter smithii DSM 2375 strain	1713248	0.31	100	1683	0
Methanobrevibacter smithii strain=ACE6	1718384	0.32	100	1694	1.87
Methanobrevibacter smithii TS145A strain=TS145A	1782572	0.31	100	1798	0
Methanobrevibacter smithii TS145B strain=TS145B	1797373	0.31	99.07	1887	0
Methanobrevibacter smithii TS146A strain=TS146A	1791997	0.31	99.07	1858	0
Methanobrevibacter smithii TS146B strain=TS146B	1794702	0.31	100	1821	0.93
Methanobrevibacter smithii TS146C strain=TS146C	1947483	0.31	92.52	2384	16.8
Methanobrevibacter smithii TS146D strain=TS146D	1713264	0.31	93.46	1698	0
Methanobrevibacter smithii TS146E strain=TS146E	1952171	0.3	96.26	1925	3.74
Methanobrevibacter smithii TS147A strain=TS147A	2008979	0.3	100	2005	6.54
Methanobrevibacter smithii TS147B strain=TS147B	1965064	0.3	99.07	1921	0
Methanobrevibacter smithii TS147C strain=TS147C	1973030	0.3	99.93	2031	0.93
Methanobrevibacter smithii TS94A strain=TS94A	1889378	0.3	100	1818	0
Methanobrevibacter smithii TS94B strain=TS94B	1886020	0.3	99.07	1872	0
Methanobrevibacter smithii TS94C strain=TS94C	1910054	0.3	100	1824	0.93
Methanobrevibacter smithii TS95A strain=TS95A	1992157	0.3	99.07	1985	0.93
Methanobrevibacter smithii TS95B strain=TS95B	1972498	0.3	99.07	1906	0
Methanobrevibacter smithii TS95C strain=TS95C	1978848	0.3	99.07	1877	0
Methanobrevibacter smithii TS95D strain=TS95D	2011683	0.3	96.26	1883	8.41
Methanobrevibacter smithii TS96A strain=TS96A	1975004	0.3	100	1859	0
Methanobrevibacter smithii TS96B strain=TS96B	1869210	0.3	96.26	1763	0.93
Methanobrevibacter smithii TS96C strain=TS96C	1818239	0.31	100	1818	0
Methanobrevibacter sp. AbM4 strain=AbM4	1998189	0.29	100	1702	0
Methanobrevibacter wolinii SH strain=SH	2041814	0.24	100	1704	0
Methanocaldococcus fervens AG86 strain=AG86	1485061	0.32	100	1604	0
Methanocaldococcus infernus ME strain=ME	1328194	0.34	99.07	1474	0
Methanocaldococcus jannaschii DSM 2661	1739927	0.31	100	1877	0
Methanocaldococcus sp. FS406-22 strain=FS406-22	1760939	0.32	100	1862	0
Methanocaldococcus sp. JH146 strain=JH146	1607556	0.31	100	1676	0
Methanocaldococcus villosus KIN24-T80 strain=KIN24-T80	1207361	0.29	92.99	1439	0
Methanocaldococcus vulcanius M7 strain=M7	1746329	0.31	100	1746	0
Methanocella arvoryzae MRE50 strain=MRE50	3179916	0.55	100	3117	0
Methanocella conradii HZ254 strain=HZ254	2378438	0.53	100	2514	0
Methanocella paludicola SANAE strain=SANAE	2957635	0.55	100	3055	0.93
Methanococcoides burtonii DSM 6242 strain=DSM 6242	2575032	0.41	99.07	2616	0
Methanococcoides methylutens MM1 strain=MM1	2394636	0.44	99.07	2328	0
Methanococcoides methylutens strain=DSM 2657	2508511	0.42	99.07	2464	0.93
Methanococcus aeolicus Nankai-3 strain=Nankai-3	1569500	0.3	100	1525	0.93
Methanococcus maripaludis C5 strain=C5	1780761	0.33	100	1879	0
Methanococcus maripaludis C6 strain=C6	1744193	0.33	100	1869	0
Methanococcus maripaludis C7 strain=C7	1772694	0.33	100	1833	0
Methanococcus maripaludis S2 strain=S2	1661137	0.33	100	1749	0
Methanococcus maripaludis X1 strain=X1	1746697	0.33	100	1869	0
Methanococcus vannielii SB strain=SB	1720048	0.31	100	1746	0.93

Methanococcus voltae A3 strain=A3	1936387	0.29	99.53	1727	2.8
Methanocorpusculum bavaricum DSM 4179	1706782	0.51	99.22	1747	0
Methanocorpusculum labreanum Z strain=Z	1804962	0.5	99.22	1816	0
Methanoculleus bourgensis MS2 strain=MS2T	2789774	0.61	99.53	2674	0
Methanoculleus bourgensis MS2 strain=type strain:MS2	2789773	0.61	99.53	2672	0.65
Methanoculleus chikugoensis JCM 10825 strain=JCM 10825	2627540	0.62	77.65	4374	1.87
Methanoculleus marisnigri JR1 strain=JR1	2478101	0.62	100	2481	0.93
Methanoculleus sp. CAG:1088	1633002	0.56	99.07	1645	0.93
Methanoculleus sp. MH98A strain=MH98A	2542436	0.61	98.6	2802	3.81
Methanoculleus sp. S3Fa strain=S3Fa	2489717	0.62	99.53	2474	0.65
Methanofollis liminatans DSM 4140 strain=DSM 4140	2475100	0.61	98.13	2438	0.93
Methanogenium cariaci JCM 10550 strain=JCM 10550	2686643	0.52	90.5	4256	0
Methanohalobium evestigatum Z-7303 strain=Z-7303	2242317	0.37	99.07	2281	1.96
Methanohalophilus mahii DSM 5219 strain=DSM 5219	2012424	0.43	97.2	2012	1.87
Methanolacinia paynteri strain=DSM 2545	2791704	0.48	98.6	2770	0.93
Methanolacinia petrolearia DSM 11571	2843290	0.47	99.53	2821	0.93
Methanolinea tarda NOBI-1 strain=NOBI-1	2052856	0.57	99.53	2064	0
Methanolobus psychrophilus R15 strain=R15	3072769	0.45	99.07	3004	0.93
Methanolobus tindarius DSM 2278 strain=DSM 2278	3151883	0.4	99.07	2955	0
Methanomassiliicoccus luminyensis B10 strain=B10	2620233	0.6	99.07	2607	0
Methanomethylovorans hollandica DSM 15978 strain	2428904	0.43	98.13	2378	0.33
Methanomicrobium mobile BP strain=BP	1711791	0.49	100	1630	0
Methanoplanus limicola DSM 2279 strain=DSM 2279	3200946	0.42	99.07	3049	2.8
Methanopyrus kandleri AV19 strain=AV19	1694969	0.61	99.93	1814	0.93
Methanoregula boonei 6A8 strain=6A8	2542943	0.55	99.22	2527	0.93
Methanoregula formicica SMSP strain=SMSP	2820858	0.55	99.69	2880	0
Methanosaeta concilii GP6 strain=GP6	3008626	0.51	99.07	3032	0.65
Methanosaeta harundinacea 6Ac strain=6Ac	2559043	0.61	100	2468	0
Methanosaeta thermophila PT strain=PT	1879471	0.54	99.07	1810	0
Methanosalsum zhilinae DSM 4017 strain=DSM 4017	2138444	0.39	100	2027	0.93
Methanosarcina acetivorans C2A strain=C2A	5751492	0.43	98.13	4884	0.93
Methanosarcina barkeri 227 strain=227	4447980	0.39	98.13	3716	0
Methanosarcina barkeri 3 strain=3	4560446	0.39	98.13	3766	0
Methanosarcina barkeri CM1 strain=CM1	4501171	0.39	98.13	3804	0
Methanosarcina barkeri JCM 10043 strain=JCM 10043	4458961	0.39	93.54	5757	0
Methanosarcina barkeri MS strain=MS	4533209	0.39	98.13	3805	0.33
Methanosarcina barkeri str. Fusaro strain=Fusaro	4837408	0.39	98.13	4016	0.65
Methanosarcina barkeri str. Wiesmoor	4874815	0.39	98.13	4041	0.65
Methanosarcina horonobensis strain=HB-1	5018607	0.41	99.07	4415	0
Methanosarcina horonobensis strain=JCM 15518	4926839	0.41	90.27	7244	1.87
Methanosarcina lacustris Z-7289 strain=Z-7289	4139808	0.42	98.13	3469	0.93
Methanosarcina mazei C16 strain=C16	4166241	0.41	99.07	3568	0
Methanosarcina mazei Go1 strain=Go1	4096345	0.41	99.07	3477	0
Methanosarcina mazei JCM 9314 strain=JCM 9314	4223846	0.42	90.82	5825	1.87
Methanosarcina mazei LYC strain=LYC	4154344	0.41	99.07	3567	0
Methanosarcina mazei S-6 strain=S-6	4142816	0.41	99.07	3558	0
Methanosarcina mazei SarPi strain=SarPi	4066551	0.41	99.07	3445	0
Methanosarcina mazei strain=1.F.A.1A.3	4049464	0.42	99.07	3482	0
Methanosarcina mazei strain=1.F.A.1B.3	4034156	0.42	99.07	3480	0
Methanosarcina mazei strain=1.F.A.1B.4	4053560	0.42	99.07	3518	0.93

Methanosarcina mazei strain=1.F.A.2.8	3971797	0.42	99.07	3430	0
Methanosarcina mazei strain=1.F.M.0.5	4072647	0.42	99.07	3520	0
Methanosarcina mazei strain=1.H.A.0.1	3968247	0.42	99.07	3426	0
Methanosarcina mazei strain=1.H.A.1A.1	4044897	0.42	99.07	3493	0
Methanosarcina mazei strain=1.H.A.1A.3	4084966	0.42	99.07	3550	0
Methanosarcina mazei strain=1.H.A.1A.4	3989219	0.42	99.07	3446	0
Methanosarcina mazei strain=1.H.A.1A.6	4079653	0.42	99.07	3552	0.93
Methanosarcina mazei strain=1.H.A.2.1	3998326	0.42	99.07	3477	0
Methanosarcina mazei strain=1.H.A.2.3	4091699	0.42	99.07	3537	0
Methanosarcina mazei strain=1.H.A.2.6	4006860	0.42	99.07	3466	0
Methanosarcina mazei strain=1.H.A.2.7	4234097	0.41	99.07	3820	0
Methanosarcina mazei strain=1.H.A.2.8	4077516	0.42	99.07	3541	0
Methanosarcina mazei strain=1.H.M.0.1	4064093	0.42	99.07	3505	0
Methanosarcina mazei strain=1.H.M.1A.1	4085382	0.42	99.07	3526	0
Methanosarcina mazei strain=1.H.M.1A.2	4081225	0.42	99.07	3517	0
Methanosarcina mazei strain=1.H.M.1A.3	4081659	0.42	99.07	3574	0
Methanosarcina mazei strain=1.H.M.2.1	4188559	0.42	99.07	3645	0
Methanosarcina mazei strain=1.H.M.2.2	4082602	0.42	99.07	3523	0
Methanosarcina mazei strain=1.H.M.2.3	3978804	0.42	99.07	3427	0
Methanosarcina mazei strain=1.H.M.2.4	4079204	0.42	99.07	3567	0
Methanosarcina mazei strain=1.H.T.2.1	4077567	0.42	99.07	3513	0
Methanosarcina mazei strain=1.H.T.2.3	3972119	0.42	99.07	3423	0
Methanosarcina mazei strain=1.H.T.2.5	4085981	0.42	99.07	3526	0
Methanosarcina mazei strain=2.F.A.2.3	4077945	0.42	99.07	3530	0
Methanosarcina mazei strain=2.F.A.2.4	4198899	0.42	99.07	3635	0
Methanosarcina mazei strain=2.F.T.0.2	4092300	0.42	99.07	3557	0
Methanosarcina mazei strain=2.F.T.2.6	4061965	0.42	99.07	3564	0
Methanosarcina mazei strain=3.F.A.1A.1	4102484	0.42	99.07	3668	0.93
Methanosarcina mazei strain=3.F.A.1A.3	4073967	0.42	99.07	3511	0
Methanosarcina mazei strain=3.F.A.1B.1	4078419	0.42	99.07	3512	0
Methanosarcina mazei strain=3.F.A.2.12	4076107	0.42	99.07	3557	0
Methanosarcina mazei strain=3.F.A.2.3	4078931	0.42	99.07	3539	0
Methanosarcina mazei strain=3.F.A.2.5	4068199	0.42	99.07	3505	0
Methanosarcina mazei strain=3.F.A.2.6	4043214	0.42	99.07	3494	0
Methanosarcina mazei strain=3.F.A.2.7	4028869	0.42	98.13	3471	0
Methanosarcina mazei strain=3.F.T.1A.1	4164852	0.41	99.07	3603	0
Methanosarcina mazei strain=3.F.T.1A.2	4157971	0.41	99.07	3594	0
Methanosarcina mazei strain=3.F.T.1A.4	4160506	0.41	99.07	3587	0
Methanosarcina mazei strain=3.F.T.2.1	4110215	0.41	99.07	3547	0
Methanosarcina mazei strain=3.H.A.1A.1	4011068	0.42	97.66	3599	0
Methanosarcina mazei strain=3.H.A.1A.2	4095685	0.42	99.07	3558	0
Methanosarcina mazei strain=3.H.A.2.1	3981997	0.42	99.07	3443	0
Methanosarcina mazei strain=3.H.A.2.4	4123985	0.41	99.07	3561	0
Methanosarcina mazei strain=3.H.A.2.5	4094146	0.42	99.07	3539	0
Methanosarcina mazei strain=3.H.A.2.6	4014381	0.42	99.07	3436	0
Methanosarcina mazei strain=3.H.A.2.8	4005945	0.42	99.07	3464	0
Methanosarcina mazei strain=3.H.M.1A.1	4121503	0.41	99.07	3555	0
Methanosarcina mazei strain=3.H.M.1B.1	4129685	0.41	99.07	3570	0
Methanosarcina mazei strain=3.H.M.1B.2	4126043	0.41	99.07	3528	0
Methanosarcina mazei strain=3.H.M.1B.5	4122823	0.41	99.07	3528	0

Methanosarcina mazei strain=3.H.M.2.7	4003619	0.42	99.07	3430	0
Methanosarcina mazei strain=3.H.T.1A.1	4075275	0.42	99.07	3515	0
Methanosarcina mazei strain=3.H.T.1A.2	4091268	0.42	99.07	3552	0
Methanosarcina mazei Tuc01 strain=Tuc01	3427949	0.42	97.66	3159	0
Methanosarcina mazei WWM610 strain=WWM610	4096482	0.41	99.07	3486	0
Methanosarcina siciliae C2J strain=C2J	5427890	0.43	98.13	4706	0
Methanosarcina siciliae HI350 strain=HI350	4941115	0.43	98.13	4178	0
Methanosarcina siciliae T4/M strain=T4/M	5017558	0.43	98.13	4232	0.93
Methanosarcina soligelidi strain=SMA-21	4064496	0.42	99.07	3461	0
Methanosarcina sp. 1.H.A.2.2 strain=1.H.A.2.2	3870729	0.43	98.13	3233	0.93
Methanosarcina sp. 1.H.T.1A.1 strain=1.H.T.1A.1	3842140	0.43	98.13	3284	1.47
Methanosarcina sp. 2.H.A.1B.4 strain=2.H.A.1B.4	3907151	0.43	98.13	3307	0.93
Methanosarcina sp. 2.H.T.1A.15 strain=2.H.T.1A.15	4080053	0.43	98.13	3639	3.27
Methanosarcina sp. 2.H.T.1A.3 strain=2.H.T.1A.3	4083606	0.43	98.13	3477	1.4
Methanosarcina sp. 2.H.T.1A.6 strain=2.H.T.1A.6	4074625	0.43	98.13	3446	1.4
Methanosarcina sp. 2.H.T.1A.8 strain=2.H.T.1A.8	4069899	0.43	98.13	3451	1.4
Methanosarcina sp. 795	3122414	0.41	98.13	2719	0.65
Methanosarcina sp. E03.2 strain=E03.2	3268085	0.41	98.13	2876	0
Methanosarcina sp. Kolksee strain=Kolksee	4499114	0.4	99.07	3702	0
Methanosarcina sp. MTP4 strain=MTP4	4211468	0.46	98.13	3520	0
Methanosarcina sp. WH1 strain=WH1	3914091	0.42	98.13	3449	0
Methanosarcina sp. WWM596 strain=WWM596	4137337	0.42	98.13	3668	0
Methanosarcina thermophila CHTI-55 strain=CHTI-55	3130228	0.41	98.13	2666	0
Methanosarcina thermophila TM-1 strain=TM-1	3127379	0.41	98.13	2712	0
Methanosarcina vacuolata Z-761 strain=Z-761	4505752	0.4	98.13	3697	1.87
Methanosphaera stadtmanae DSM 3091 strain	1767403	0.28	99.07	1552	0.93
Methanosphaerula palustris E1-9c strain=E1-9c	2922917	0.55	99.53	2779	0.93
Methanospirillum hungatei JF-1 strain=JF-1	3544738	0.45	98.6	3415	0
Methanothermobacter marburgensis str. Marburg	1634695	0.49	100	1744	0.93
Methanothermobacter sp. CaT2 strain=CaT2	1720003	0.49	100	1777	0
Methanothermobacter thermautotrophicus str. Delta H	1751377	0.5	100	1876	0.36
Methanothermococcus okinawensis IH1 strain=IH1	1662525	0.29	100	1655	0
Methanothermococcus thermolithotrophicus DSM 2095	1686930	0.33	100	1704	0.93
Methanothermus fervidus DSM 2088 strain=DSM 2088	1243342	0.32	100	1318	0
Methanotorris formicicus Mc-S-70 strain=Mc-S-70	1818783	0.32	100	2045	0
Methanotorris igneus Kol 5 strain=Kol 5	1854197	0.32	100	1822	0.93
Methermicoccus shengliensis DSM 18856 strain	1513583	0.55	100	1612	0
Palaeococcus ferrophilus DSM 13482 strain=DSM 13482	2206431	0.54	100	2350	0
Palaeococcus pacificus DY20341 strain=DY20341	1859370	0.43	100	1991	0
Picrophilus torridus DSM 9790 strain=DSM 9790	1545895	0.36	98.6	1599	0.93
Pyrococcus abyssi GE5	1765118	0.45	100	1913	0
Pyrococcus furiosus COM1 strain=COM1	1909827	0.41	99.77	2103	0
Pyrococcus furiosus DSM 3638 strain=DSM 3638	1908256	0.41	99.77	2102	0
Pyrococcus horikoshii OT3 strain=OT3	1738505	0.42	100	1881	0
Pyrococcus sp. NA2 strain=NA2	1861320	0.43	100	2005	0
Pyrococcus sp. ST04 strain=ST04	1736885	0.42	100	1831	0
Pyrococcus yayanosii CH1 strain=CH1	1716818	0.52	100	1882	0
Salinarchaeum_sp._Harcht-Bsk1	3255260	0.67	100	3105	0
Thermococcus barophilus MP strain=MP	2010078	0.42	100	2172	0
Thermococcus celer JCM 8558 strain=JCM 8558	1890146	0.57	83.2	3529	0

Thermococcus cleftensis strain=CL1	1950313	0.56	100	2074	0
Thermococcus eurythermalis strain=A501	2122535	0.53	100	2277	0
Thermococcus gammatolerans EJ3 strain=EJ3; DSM 15229	2045438	0.54	100	2175	0
Thermococcus kodakarensis KOD1 strain=KOD1	2088737	0.52	100	2314	0
Thermococcus litoralis DSM 5473 strain=DSM 5473	2215172	0.43	100	2497	0.93
Thermococcus nautili strain=30-1	1976356	0.55	100	2181	0
Thermococcus onnurineus NA1 strain=NA1	1847607	0.51	99.77	1998	0
Thermococcus paralvinellae strain=ES1	1957742	0.4	98.13	2136	0
Thermococcus peptonophilus JCM 9653 strain=JCM 9653	1909446	0.52	87.79	3302	0.04
Thermococcus sibiricus MM 739 strain=MM 739	1845800	0.4	100	2021	0
Thermococcus sp. 4557 strain=4557	2011320	0.56	100	2118	0
Thermococcus sp. AM4 strain=AM4	2086428	0.55	100	2274	0
Thermococcus sp. EP1 strain=EP1	1819157	0.39	100	1977	0
Thermococcus sp. JCM 11816 strain=JCM 11816	2113420	0.52	90.58	3717	0.93
Thermococcus sp. PK strain=PK	2176573	0.44	98.13	2424	1.87
Thermococcus zilligii AN1 strain=AN1	1764559	0.54	99.77	1869	0
Thermogymnomonas acidicola JCM 13583 strain	1742379	0.56	83.34	3240	0.93
Thermoplasma acidophilum DSM 1728 strain=DSM1728	1564906	0.46	97.35	1570	0
Thermoplasma volcanium GSS1 strain=GSS1	1584804	0.4	97.66	1635	0
Thermoplasmatales archaeon A-plasma	1989604	0.46	94.86	2277	0.93
Thermoplasmatales archaeon BRNA1 strain=BRNA1	1461105	0.58	98.6	1506	0.93
Thermoplasmatales archaeon E-plasma	1660504	0.39	96.11	1714	0
Thermoplasmatales archaeon SCGC AB-539-C06	593453	0.35	29.58	784	1.64
Thermoplasmatales archaeon SCGC AB-539-N05	801028	0.36	58.2	854	1.81
Thermoplasmatales archaeon SCGC AB-540-F20	1037251	0.36	35.51	1258	0
Candidatus Nanosalinarum sp. J07AB56	1227157	0.43	78.5	1581	3.73
Candidatus Nanosalina sp. J07AB43	1215802	0.56	75.3	1505	4.47
Candidatus_Halobonum_tyrrellensis_G22	3675087	0.7	100	3525	0.93
Haladaptatus_cibarius_D43_HAPD43	3926724	0.58	100	4125	0.93
Haladaptatus_paucihalophilus_DX253	4284805	0.62	100	4364	0.93
Haladaptatus_paucihalophilus_DX253_B208DRAFT	4317540	0.62	100	4391	0.93
Halalkalicoccus_jeotgali_B3	3637498	0.63	100	3860	0.93
Halanaeroarchaeum_sulfurireducens_strain_HSR2	2085482	0.63	100	2137	0
Halanaeroarchaeum_sulfurireducens_strain_M27-SA2	2253500	0.63	100	2307	0
Halapricum_salinum	3451492	0.64	100	3450	0
Haloarcula_californiae_ATCC_33799	4404932	0.61	100	4573	0
Haloarcula_hispanica_ATCC_33960	3484189	0.63	100	3535	0
Haloarcula_hispanica_N601	3369916	0.63	100	3423	0
Haloarcula_japonica_DSM_6131	4280359	0.61	100	4384	0.93
Haloarcula_marismortui_ATCC_43049	4274642	0.61	100	4453	0
Haloarcula_sinaiiensis_ATCC_33800	4405164	0.61	100	4543	0
Haloarcula_sp._CBA1115	3423144	0.63	100	3480	0
Haloarcula_sp._SL3	3970989	0.62	100	4056	0
Haloarcula_vallismortis_ATCC_29715	3923205	0.62	100	4060	0
Halobiforma_lacisalsi_AJ5	4338576	0.65	100	4264	0
Halococcus_hamelinensis_100A6	3419074	0.65	100	3516	0.07
Halococcus_sediminicola	3764367	0.62	100	3907	0.93
Halococcus_thailandensis_JCM_13552	4052434	0.62	100	4366	1.87
Haloferax_alexandrinus_Arc-Hr	3893626	0.66	100	3848	0
Haloferax_alexandrinus_JCM_10717	3649262	0.66	100	3666	0

Haloferax_denitrificans_ATCC_35960	3825973	0.66	100	3809	0
Haloferax_elongans_ATCC_BAA-1513	3952136	0.61	100	3994	0
Haloferax_gibbonsii_ATCC_33959	4057322	0.66	100	4022	0
Haloferax_gibbonsii_strain_ARA6	2945391	0.67	100	3034	0
Haloferax_larsenii_JCM_13917	3697948	0.62	100	3714	0
Haloferax_lucentense_DSM_14919	3619064	0.66	100	3654	0
Haloferax_prahovense_DSM_18310	3998799	0.66	100	3991	0
Haloferax_sp._Arc-Hr	4015175	0.66	100	3919	0
Haloferax_sp._ATCC_BAA-645	3598205	0.67	100	3616	0
Haloferax_sulfurifontis_ATCC_BAA-897	3812428	0.66	100	3850	0
Haloferax_volcanii_DS2	3866754	0.66	100	3931	0
Halogeometricum_borinquense_DSM_11551	2820544	0.61	100	2905	0
Halolamina_rubra	2955001	0.69	100	3123	1.25
Halolamina_sp._halo-7	2835858	0.68	100	2886	0
Halomicrobium_katesii_DSM_19301	3607771	0.65	100	3661	0.93
Halomicrobium_mukohataei_DSM_12286	3110487	0.66	100	3198	0
Halopiger_sp._IIH2	3776554	0.64	100	3719	0
Halopiger_sp._IIH3	3906364	0.66	100	3853	1.22
Halopiger_xanaduensis_SH-6	3668009	0.66	100	3601	0
Halorhabdus_tiamatea_SARL4B	2815791	0.63	100	2835	0
Halorhabdus_utahensis_DSM_12940	3116795	0.63	100	3043	0.93
Halorubrum_aidingense_JCM_13560	3108525	0.67	100	3062	0.93
Halorubrum_arcis_JCM_13916	3382601	0.67	100	3376	0
Halorubrum_coriense_DSM_10284	3645313	0.67	100	3623	0.93
Halorubrum_distributum_JCM_10118	3306135	0.68	100	3223	0
Halorubrum_distributum_JCM_9100	3307369	0.68	100	3231	0
Halorubrum_ezzemoulense_DSM_17463	3591480	0.67	100	3555	0.47
Halorubrum_halophilum	3677984	0.65	100	3670	0.93
Halorubrum_hochstenium_ATCC_700873	3037532	0.69	100	3008	0
Halorubrum_lacusprofundi_ATCC_49239	3261238	0.65	100	3235	0
Halorubrum_lipolyticum_DSM_21995	3425042	0.68	100	3308	0
Halorubrum_litoreum_JCM_13561	3137757	0.69	100	3100	0
Halorubrum_saccharovorum_DSM_1137	3423703	0.67	100	3353	0.93
Halorubrum_sp._5	3567018	0.68	100	3595	0
Halorubrum_sp._BV1	2767463	0.66	100	2765	0
Halorubrum_sp._SD626R	3248714	0.68	100	3284	3.88
Halorubrum_sp._T3	3168011	0.68	100	3054	0
Halorubrum_tebenquichense_DSM_14210	3328860	0.68	100	3354	0
Halorubrum_terrestre_JCM_10247	3376225	0.68	100	3361	0
Halosimplex_carlsbadense_2-9-1	4694889	0.68	100	4581	1.87
Halostagnicola_larsenii_XH-48	2789326	0.62	100	2871	0
Haloterrigena_jeotgali_A29_HTGA29	4131621	0.65	100	4262	0
Haloterrigena_limicola_JCM_13563	3522035	0.62	100	3657	1.87
Haloterrigena_turkmenica_DSM_5511	3889038	0.66	100	3753	0
Halovivax_asiaticus_JCM_14624	3238452	0.64	100	3215	0
Halovivax_ruber_XH-70	3223876	0.64	100	3132	0
Natrialba_asiatica_DSM_12278	4404175	0.62	100	4319	0
Natrialba_chahannaoensis_JCM_10990	4309274	0.6	100	4262	0
Natrialba_magadii_ATCC_43099	3751858	0.61	100	3622	0
Natrinema_altunense_JCM_12890	3774970	0.65	100	3814	0

Natrinema_altunense_strain_AJ2_N_altunense_AJ2	3774135	0.65	100	3794	0
Natrinema_gari_JCM_14663_	4023692	0.64	100	4149	0
Natrinema_pellirubrum_DSM_15624	4264455	0.64	100	4336	0
Natrinema_sp._J7-1	3667624	0.64	100	3732	0
Natrinema_versiforme_JCM_10478	4190799	0.64	100	4285	0
Natronococcus_amylolyticus_DSM_10524	4416525	0.64	100	4459	0.93
Natronococcus_occultus_SP4	4013216	0.65	100	4007	0
Natronolimnobius_innermongolicus_JCM_12255	4588634	0.64	100	4467	0
Natronomonas_moolapensis_8.8.11	2912573	0.65	100	2923	0
Natronomonas_pharaonis_DSM_2160	2595221	0.63	100	2679	0
Natronorubrum_bangense_JCM_10635	4111275	0.6	100	4167	0.93
Natronorubrum_sulfidifaciens_JCM_14089	3460288	0.62	100	3516	0
Natronorubrum_tibetense_GA33	4934841	0.62	100	4750	0.07
Salinarchaeum_sp._Harcht-Bsk1	3255260	0.67	100	3105	0
Halobacterium_sp._DL1	2846968	0.67	99.77	2988	0
haloarchaeon_3A1_DGR	2880902	0.68	99.53	2831	1.87
Haloarcula_amylolytica_JCM_13557	4225424	0.62	99.53	4327	0.93
Haloarcula_argentinensis_DSM_12282	4147107	0.61	99.53	4272	0
Haloferax_mediterranei_ATCC_33500	2946877	0.61	99.53	3076	0
Natrialba_aegyptia_DSM_13077	4618362	0.62	99.53	4586	0
Natrialba_hulunbeirensis_JCM_10989	4159606	0.62	99.53	3984	0.47
Natrialba_taiwanensis_DSM_12281	4635192	0.62	99.53	4604	0
Natronococcus_jeotgali_DSM_18795	4496185	0.64	99.53	4632	0.93
Natrialba_magadii_ATCC_43099_strain_MS-3	4416299	0.61	99.22	4362	0.93
Halococcus_agarilyticus	3476728	0.66	99.07	3475	0
Halococcus_morrhuae_DSM_1307	2991556	0.64	99.07	3175	0
Halococcus_saccharolyticus_DSM_5350	3449700	0.64	99.07	3582	1.87
Haloferax_mucosum_ATCC_BAA-1512	3368982	0.62	99.07	3454	0
Halogeometricum_pallidum_JCM_14848	4384515	0.66	99.07	4349	0.93
Halogranum_salarium_B-1_162.HSB1.1_1	4492306	0.62	99.07	4528	0
Halopiger_salifodinae_strain_KCY07-B2	4350898	0.65	99.07	4248	3.74
Haloplanus_natans_DSM_17983	3797935	0.65	99.07	3968	0
Haloquadratum_walsbyi_C23	3148033	0.48	99.07	2984	0
Haloquadratum_walsbyi_DSM_16790	3132494	0.48	99.07	3015	0
Haloterrigena_thermotolerans_DSM_11522	3895275	0.65	99.07	3938	0
Natrinema_pallidum_DSM_3751	3915814	0.64	99.07	3969	0.93
Natrinema_sp._J7-2	3697626	0.64	99.07	3725	0
Natronobacterium_gregoryi_SP2	3694030	0.62	99.07	3746	0
Haloferax_sp._BAB2207_V.1	3744707	0.66	98.99	4415	0.93
Halococcus_salifodinae_DSM_8989	4199784	0.63	98.6	4323	0.93
Halolamina_pelagica_strain_CDK2	2972542	0.68	98.6	3506	0.93
Haloterrigena_salina_JCM_13891	4841607	0.65	98.6	4652	0
Haloferax_sp._ATCC_BAA-644	3587033	0.67	98.13	3602	0
Halarchaeum_acidiphilum_MH1-52-1	2628974	0.67	97.35	2856	0
Halobacterium_salinarum_R1	2000962	0.68	97.2	2102	0
Halobacterium_sp._NRC-1	2014239	0.68	97.2	2106	0
Halobellus_rufus	3852219	0.64	97.2	3971	0
Halorubrum_kocurii_JCM_14978	3619738	0.67	97.2	3571	0
Halonotius_sp._J07HN6	2529000	0.63	96.42	2837	3.74
Halobiforma_nitratireducens_JCM_10879	3688747	0.64	95.33	3676	0.93

Halonotius_sp._J07HN4	2888659	0.61	94.16	3196	24.3
Halostagnicola_sp._A56	3178490	0.61	93.54	3401	1.01
Halorubrum_saccharovorum_strain_H3_NODE_1	3282373	0.66	93.39	3823	0.93
Haloquadratum_walsbyi_J07HQW2	3594539	0.48	92.14	3845	1.87
halophilic_archaeon_J07HX64	2982938	0.64	87.87	3041	2.34
Natronolimnobius_baerhuensis_JCM_12253	3893062	0.6	85.9	5302	0
Natrinema_altunense_strain_1A4-DGR	3718305	0.65	85.84	5159	0
Haloquadratum_walsbyi_J07HQW1	3475501	0.49	84.82	3534	0
Haloarcula_salaria_strain_H5-DGR	4091500	0.62	82.88	6042	0
halophilic_archaeon_J07HB67	2649547	0.67	69.7	3134	0
Haloquadratum_sp._J07HQX50	3019909	0.51	67.3	3908	0
Halorubrum_sp._AJ67_WGS	4225006	0.64	58.18	4618	0.47
Halorubrum_sp._J07HR59	2120805	0.6	58.1	3039	0
halophilic_archaeon_J07HX5	2040945	0.61	38.79	2570	2.8
Haloferax_sp._ATB1	4223705	0.62	28.97	4381	5.61
Haloterrigena_sp._H13	596279	0.65	9.262	1397	0

Supplementary Table S3 List of 111 Halobacterial genomes for final analysis

Organism Name	Genome Size	GC content	Completeness(%)	Predicted Genes	Contamination
Candidatus_Halobonum_tyrrellensis_G22	3675087	0.70118	100	3525	0.935
Haladaptatus_cibarius_D43_HAPD43	3926724	0.577559	100	4125	0.935
Haladaptatus_paucihalophilus_DX253	4284805	0.618152	100	4364	0.935
Haladaptatus_paucihalophilus_DX253_B208DRAFT	4317540	0.617664	100	4391	0.935
Halalkalicoccus_jeotgali_B3	3637498	0.626317	100	3860	0.935
Halanaeroarchaeum_sulfurireducens_strain_HSR2	2085482	0.632909	100	2137	0
Halanaeroarchaeum_sulfurireducens_strain_M27-SA2	2253500	0.627608	100	2307	0
Halapricum_salinum	3451492	0.637314	100	3450	0
Haloarcula_californiae_ATCC_33799	4404932	0.608221	100	4573	0
Haloarcula_hispanica_ATCC_33960	3484189	0.627537	100	3535	0
Haloarcula_hispanica_N601	3369916	0.629974	100	3423	0
Haloarcula_japonica_DSM_6131	4280359	0.611769	100	4384	0.935
Haloarcula_marismortui_ATCC_43049	4274642	0.611188	100	4453	0
Haloarcula_sinaiiensis_ATCC_33800	4405164	0.607707	100	4543	0
Haloarcula_sp._CBA1115	3423144	0.629699	100	3480	0
Haloarcula_sp._SL3	3970989	0.620487	100	4056	0
Haloarcula_vallismortis_ATCC_29715	3923205	0.617976	100	4060	0
Halobiforma_lacisalsi_AJ5	4338576	0.653306	100	4264	0
Halococcus_hamelinensis_100A6	3419074	0.653983	100	3516	0.072
Halococcus_sediminicola	3764367	0.622933	100	3907	0.935
Halococcus_thailandensis_JCM_13552	4052434	0.618221	100	4366	1.869
Haloferax_alexandrinus_Arc-Hr	3893626	0.660046	100	3848	0
Haloferax_alexandrinus_JCM_10717	3649262	0.663011	100	3666	0
Haloferax_denitrificans_ATCC_35960	3825973	0.662868	100	3809	0
Haloferax_elongans_ATCC_BAA-1513	3952136	0.611545	100	3994	0
Haloferax_gibbonsii_ATCC_33959	4057322	0.658549	100	4022	0
Haloferax_gibbonsii_strain_ARA6	2945391	0.670617	100	3034	0
Haloferax_larsenii_JCM_13917	3697948	0.620364	100	3714	0
Haloferax_lucentense_DSM_14919	3619064	0.663902	100	3654	0
Haloferax_prahovense_DSM_18310	3998799	0.656898	100	3991	0
Haloferax_sp._Arc-Hr	4015175	0.663407	100	3919	0
Haloferax_sp._ATCC_BAA-645	3598205	0.666457	100	3616	0
Haloferax_sulfurifontis_ATCC_BAA-897	3812428	0.663011	100	3850	0
Haloferax_volcanii_DS2	3866754	0.65767	100	3931	0
Halogeometricum_borinquense_DSM_11551	2820544	0.610636	100	2905	0
Halolamina_rubra	2955001	0.69048	100	3123	1.246
Halolamina_sp._halo-7	2835858	0.67943	100	2886	0
Halomicrobium_katesii_DSM_19301	3607771	0.650319	100	3661	0.935
Halomicrobium_mukohataei_DSM_12286	3110487	0.656291	100	3198	0
Halopiger_sp._IIH2	3776554	0.643095	100	3719	0
Halopiger_sp._IIH3	3906364	0.660643	100	3853	1.222
Halopiger_xanaduensis_SH-6	3668009	0.659818	100	3601	0

Halorhabdus_tiamatea_SARL4B	2815791	0.633686	100	2835	0
Halorhabdus_utahensis_DSM_12940	3116795	0.629	100	3043	0.935
Halorubrum_aidingense_JCM_13560	3108525	0.671906	100	3062	0.935
Halorubrum_arcis_JCM_13916	3382601	0.673367	100	3376	0
Halorubrum_coriense_DSM_10284	3645313	0.669931	100	3623	0.935
Halorubrum_distributum_JCM_10118	3306135	0.680821	100	3223	0
Halorubrum_distributum_JCM_9100	3307369	0.68075	100	3231	0
Halorubrum_ezzemoulense_DSM_17463	3591480	0.666031	100	3555	0.467
Halorubrum_halophilum	3677984	0.65139	100	3670	0.935
Halorubrum_hochstenium_ATCC_700873	3037532	0.691305	100	3008	0
Halorubrum_lacusprofundi_ATCC_49239	3261238	0.65177	100	3235	0
Halorubrum_lipolyticum_DSM_21995	3425042	0.680129	100	3308	0
Halorubrum_litoreum_JCM_13561	3137757	0.689038	100	3100	0
Halorubrum_saccharovorum_DSM_1137	3423703	0.668851	100	3353	0.935
Halorubrum_sp._5	3567018	0.676464	100	3595	0
Halorubrum_sp._BV1	2767463	0.660097	100	2765	0
Halorubrum_sp._SD626R	3248714	0.675605	100	3284	3.882
Halorubrum_sp._T3	3168011	0.684782	100	3054	0
Halorubrum_tebenquichense_DSM_14210	3328860	0.680335	100	3354	0
Halorubrum_terrestre_JCM_10247	3376225	0.680296	100	3361	0
Halosimplex_carlsbadense_2-9-1	4694889	0.676748	100	4581	1.869
Halostagnicola_larsenii_XH-48	2789326	0.619472	100	2871	0
Haloterrigena_jeotgali_A29_HTGA29	4131621	0.649187	100	4262	0
Haloterrigena_limicola_JCM_13563	3522035	0.61796	100	3657	1.869
Haloterrigena_turkmenica_DSM_5511	3889038	0.658334	100	3753	0
Halovivax_asiaticus_JCM_14624	3238452	0.644544	100	3215	0
Halovivax_ruber_XH-70	3223876	0.64336	100	3132	0
Natrialba_asiatica_DSM_12278	4404175	0.62389	100	4319	0
Natrialba_chahannaoensis_JCM_10990	4309274	0.604176	100	4262	0
Natrialba_magadii_ATCC_43099	3751858	0.614208	100	3622	0
Natrinema_altunense_JCM_12890	3774970	0.645492	100	3814	0
Natrinema_altunense_strain_AJ2_N_altunense_AJ2	3774135	0.645583	100	3794	0
Natrinema_gari_JCM_14663_	4023692	0.637226	100	4149	0
Natrinema_pellirubrum_DSM_15624	4264455	0.641246	100	4336	0
Natrinema_sp._J7-1	3667624	0.643755	100	3732	0
Natrinema_versiforme_JCM_10478	4190799	0.639569	100	4285	0
Natronococcus_amylolyticus_DSM_10524	4416525	0.643628	100	4459	0.935
Natronococcus_occultus_SP4	4013216	0.649358	100	4007	0
Natronolimnobius_innermongolicus_JCM_12255	4588634	0.642905	100	4467	0
Natronomonas_moolapensis_8.8.11	2912573	0.645258	100	2923	0
Natronomonas_pharaonis_DSM_2160	2595221	0.634402	100	2679	0
Natronorubrum_bangense_JCM_10635	4111275	0.603912	100	4167	0.935
Natronorubrum_sulfidifaciens_JCM_14089	3460288	0.617798	100	3516	0
Natronorubrum_tibetense_GA33	4934841	0.62254	100	4750	0.072
Salinarchaeum_sp._Harcht-Bsk1	3255260	0.66586	100	3105	0
Halobacterium_sp._DL1	2846968	0.67485	99.8	2988	0
haloarchaeon_3A1_DGR	2880902	0.67681	99.5	2831	1.869
Haloarcula_amylolytica_JCM_13557	4225424	0.621272	99.5	4327	0.935
Haloarcula_argentinensis_DSM_12282	4147107	0.611161	99.5	4272	0
Haloferax_mediterranei_ATCC_33500	2946877	0.611317	99.5	3076	0

Natrialba_aegyptia_DSM_13077	4618362	0.619845	99.5	4586	0
Natrialba_hulunbeirensis_JCM_10989	4159606	0.617277	99.5	3984	0.467
Natrialba_taiwanensis_DSM_12281	4635192	0.615212	99.5	4604	0
Natronococcus_jeotgali_DSM_18795	4496185	0.644111	99.5	4632	0.935
Natrialba_magadii_ATCC_43099_strain_MS-3	4416299	0.610546	99.2	4362	0.935
Halococcus_agarilyticus	3476728	0.660237	99.1	3475	0
Halococcus_morrhuae_DSM_1307	2991556	0.63845	99.1	3175	0
Halococcus_saccharolyticus_DSM_5350	3449700	0.640286	99.1	3582	1.869
Haloferax_mucosum_ATCC_BAA-1512	3368982	0.618477	99.1	3454	0
Halogeometricum_pallidum_JCM_14848	4384515	0.656457	99.1	4349	0.935
Halogranum_salarium_B-1_162.HSB1.1_1	4492306	0.622007	99.1	4528	0
Halopiger_salifodinae_strain_KCY07-B2	4350898	0.654137	99.1	4248	3.738
Haloplanus_natans_DSM_17983	3797935	0.650076	99.1	3968	0
Haloquadratum_walsbyi_C23	3148033	0.477826	99.1	2984	0
Haloquadratum_walsbyi_DSM_16790	3132494	0.478639	99.1	3015	0
Haloterrigena_thermotolerans_DSM_11522	3895275	0.653889	99.1	3938	0
Natrinema_pallidum_DSM_3751	3915814	0.637196	99.1	3969	0.935
Natrinema_sp._J7-2	3697626	0.64249	99.1	3725	0
Natronobacterium_gregoryi_SP2	3694030	0.622999	99.1	3746	0

Supplementary Figure 1 Curve of pan and core genes versus number of genomes (Euryarchaeota)

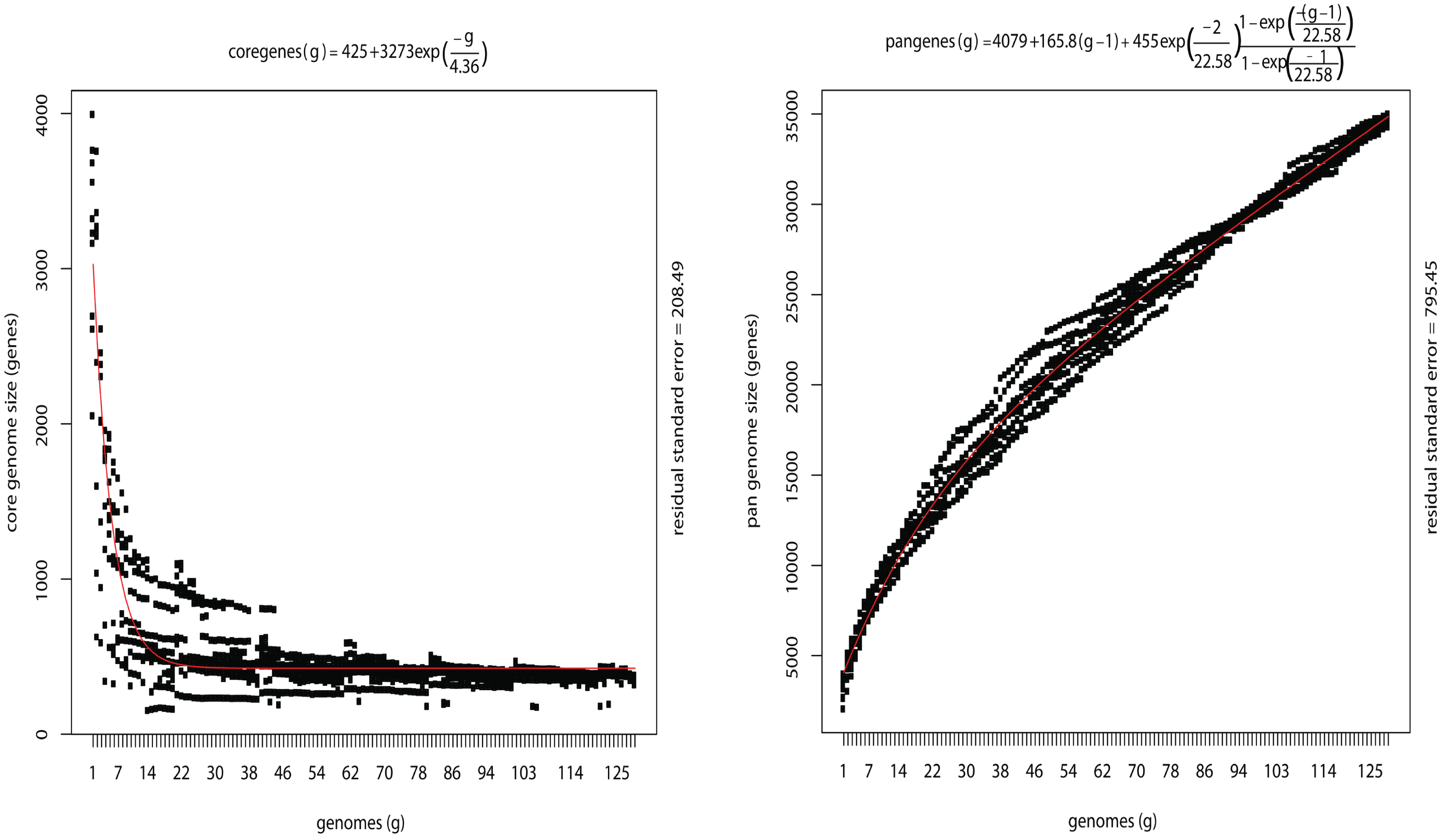

(A) (B)

Supplementary Table S5 List of gene family gain, loss and present families at the nodes of the ML tree

node	family_gain	family_loss	families
Methanopyrus_kandleri_AV19	842	275	1744
Methanothermobacter_thermautotrophicus_str._Delta_H	600	61	1762
Archaeoglobus_fulgidus_DSM_4304	851	84	2280
Haloquadratum_walsbyi_DSM_16790	543	59	2927
Thermococcus_kodakarensis_KOD1	743	38	2133
Methanocella_paludicola_SANAE	1327	119	2585
Haloarcula_marismortui_ATCC_43049	423	142	4046
Pyrococcus_horikoshii_OT3	411	84	1755
Methanococcus_maripaludis_S2	422	67	1624
Methanoculleus_marisnigri_JR1	970	151	2166
Methanobrevibacter_smithii_ATCC_35061	583	164	1642
Halorubrum_lacusprofundi_ATCC_49239	632	206	3229
Halorhabdus_utahensis_DSM_12940	465	150	2790
Halomicrobium_mukohataei_DSM_12286	307	120	3142
Methanocaldococcus_vulcanius_M7	504	119	1654
Haloterrigena_turkmenica_DSM_5511	1186	349	4421
Ferroglobus_placidus_DSM_10642	938	76	2375
Natrialba_magadii_ATCC_43099	35	11	3863
Aciduliprofundum_boonei_T469	216	24	1476
Haloferax_volcanii_DS2	399	122	3624
Natronomonas_pharaonis_DSM_2160	573	299	2588
Haladaptatus_paucihalophilus_DX253	905	141	3781
Halalkalicoccus_jeotgali_B3	1187	456	3418
Halorhabdus_tiamatea_SARL4B	787	337	2925
Halopiger_xanaduensis_SH-6	794	393	3770
Candidatus_Nanosalinarum_sp._J07AB56	874	134	1416
Candidatus_Nanosalina_sp._J07AB43	950	120	1506
Haloarcula_hispanica_ATCC_33960	2	8	3584
Halobiforma_lacisalsi_AJ5	928	207	3767
Natronobacterium_gregoryi_SP2	822	536	3332
Natrinema_pellirubrum_DSM_15624	578	157	3712
Halobacterium_sp._DL1	826	221	2942
Haloquadratum_walsbyi_C23	508	63	2888
Natrinema_sp._J7-2	40	12	3444
Halogranum_salarium_B-1	1211	260	3913
Halorubrum_sp._T3	285	226	2851
Candidatus_Methanomethylophilus_alvus_Mx1201	618	258	1502
Methanomassiliicoccus_luminyensis_B10	1183	47	2278
Aciduliprofundum_sp._MAR08-339	176	23	1437
Halovivax_ruber_XH-70	334	119	2851
Natronococcus_occultus_SP4	528	312	3723
Halococcus_sp._197A	667	233	3115
Haloarcula_amylolytica_JCM_13557	508	128	3913
Haloarcula_japonica_DSM_6131	670	277	3925
Halococcus_hamelinensis_100A6	774	339	3111

Halococcus_morrhuae_DSM_1307	414	303	2829
Halococcus_thailandensis_JCM_13552	1145	156	3707
Haloferax_alexandrinus_JCM_10717	163	61	3400
Haloferax_elongans_ATCC_BAA-1513	571	98	3654
Haloferax_gibbonsii_ATCC_33959	315	76	3667
Haloferax_lucentense_DSM_14919	164	93	3369
Haloferax_prahovense_DSM_18310	288	81	3611
Haloferax_sp._ATCC_BAA-645	28	21	3326
Haloferax_sp._ATCC_BAA-646	42	26	3335
Haloarcula_argentinensis_DSM_12282	538	209	3891
Halococcus_saccharolyticus_DSM_5350	752	185	3248
Haloferax_larsenii_JCM_13917	349	106	3424
Halorubrum_aidingense_JCM_13560	525	234	2833
Halorubrum_arcis_JCM_13916	431	139	3086
Halorubrum_coriense_DSM_10284	641	200	3260
Halorubrum_distributum_JCM_9100	37	47	2962
Halorubrum_hochstenium_ATCC_700873	245	167	2806
Halogeometricum_pallidum_JCM_14848	1110	270	3790
Haloterrigena_thermotolerans_DSM_11522	300	141	3519
Natrialba_chahannaoensis_JCM_10990	756	358	3792
Natrinema_altunense_JCM_12890	63	28	3469
Natrinema_gari_JCM_14663	540	72	3687
Natrinema_versiforme_JCM_10478	904	334	3773
Natronolimnobius_innermongolicus_JCM_12255	818	603	3799
Natronorubrum_tibetense_GA33	972	249	4108
Haloarcula_sinaiiensis_ATCC_33800	466	163	4068
Haloferax_mediterranei_ATCC_33500	684	159	3582
Halorubrum_distributum_JCM_10118	30	42	2959
Halorubrum_lipolyticum_DSM_21995	529	262	3054
Halorubrum_litoreum_JCM_13561	273	134	2893
Halorubrum_tebenquichense_DSM_14210	461	134	3055
Halorubrum_terrestre_JCM_10247	354	77	3031
Halosimplex_carlsbadense_2-9-1	1373	265	4036
Haloterrigena_limicola_JCM_13563	652	543	3253
Halovivax_asiaticus_JCM_14624	430	129	2937
Natrialba_aegyptia_DSM_13077	613	175	4029
Natrialba_asiatica_DSM_12278	540	212	3815
Natrialba_hulunbeirensis_JCM_10989	470	293	3571
Natrialba_taiwanensis_DSM_12281	706	250	4047
Natrinema_pallidum_DSM_3751	671	251	3581
Natronococcus_amylolyticus_DSM_10524	585	304	3811
Natronococcus_jeotgali_DSM_18795	915	480	3942
Natronorubrum_bangense_JCM_10635	687	205	3648
Natronorubrum_sulfidifaciens_JCM_14089	361	354	3173
Haloarcula_californiae_ATCC_33799	470	108	4087
Haloarcula_vallismortis_ATCC_29715	513	195	3732
Haloferax_denitrificans_ATCC_35960	250	108	3453
Haloferax_mucosum_ATCC_BAA-1512	408	269	3196
Haloferax_sulfurifontis_ATCC_BAA-897	395	185	3521
Halogeometricum_borinquense_DSM_11551	971	307	3614

Natrialba_magadii_ATCC_43099	95	28	3906
Halorubrum_saccharovorum_DSM_1137	613	215	3097
Halomicrobium_katesii_DSM_19301	503	141	3317
Salinarchaeum_sp._Harcht-Bsk1	755	526	2891
Halorubrum_ezzemoulense_DSM_17463	556	185	3190
Haloplanus_natans_DSM_17983	1348	595	3596
haloarchaeon_3A1_DGR	618	525	2613
Halopiger_goleamassiliensis_IIH3	667	275	3479
Halopiger_djelfamassiliensis_IIH2	653	383	3357
Natrinema_sp._J7-1	36	87	3365
Candidatus_Halobonum_tyrrellensis_G22	932	380	3141
Haloarcula_hispanica_N601	4	1	3593
Halostagnicola_larsenii_XH-48	826	372	3526
Natronomonas_moolapensis_8.8.11	631	277	2668
Haloterrigena_jeotgali_A29	557	91	3826
Haladaptatus_cibarius_D43	909	228	3698
Haloferax_alexandrinus_Arc-Hr	182	52	3534
Natrinema_altunense_AJ2	29	14	3449
Halolamina_rubra_CBA1107	654	248	2881
Halorubrum_halophilum_B8	695	216	3266
Halorubrum_sp._BV1	457	433	2566
Halapricum_salinum_CBA1105	824	281	3166
Halococcus_sediminicola_CBA1101	780	138	3408
Halopiger_salifodinae_KCY07-B2	738	434	3706
Haloarcula_sp._CBA1115	382	79	3867
Halanaeroarchaeum_sulfurireducens_HSR2	12	4	2116
Haloferax_gibbonsii_ARA6	209	92	3545
Halorubrum_sp._SD626R	562	300	2950
Haloarcula_sp._SL3	428	152	3690
Halorubrum_sp._5	578	184	3238
Halolamina_sp._halo-7	424	221	2678
Halanaeroarchaeum_sulfurireducens_M27-SA2	62	3	2167
Haloferax_sp._Arc-Hr	319	170	3572
$128_noname	525	274	1428
$129_noname	29	90	1177
$130_noname	149	138	1223
$131_noname	176	119	1269
$132_noname	68	94	1212
$133_noname	82	61	1238
$134_noname	434	274	1284
$135_noname	172	154	1142
$136_noname	95	188	1124
$137_noname	27	106	1217
$138_noname	374	157	1513
$139_noname	105	156	1296
$140_noname	79	109	1347
$141_noname	361	133	1377
$142_noname	564	990	2443
$143_noname	198	117	2950
$144_noname	51	96	2869

$145_noname	59	108	3298
$146_noname	85	27	3347
$147_noname	147	117	3319
$148_noname	84	151	3289
$149_noname	78	123	3311
$150_noname	22	89	3356
$151_noname	125	72	3423
$152_noname	53	44	3428
$153_noname	48	63	3404
$154_noname	119	70	3419
$155_noname	323	132	3370
$156_noname	108	230	3057
$157_noname	106	60	3179
$158_noname	270	222	3181
$159_noname	344	125	3133
$160_noname	122	170	2914
$161_noname	221	102	2962
$162_noname	138	43	2843
$163_noname	94	110	2787
$164_noname	145	41	2803
$165_noname	113	102	2699
$166_noname	46	51	2688
$167_noname	13	164	2542
$168_noname	53	50	2693
$169_noname	48	265	2754
$170_noname	3	4	2971
$171_noname	264	86	2972
$172_noname	99	97	2794
$173_noname	71	123	2792
$174_noname	58	76	2844
$175_noname	65	108	2819
$176_noname	90	28	2862
$177_noname	115	187	2728
$178_noname	175	65	2800
$179_noname	301	131	2690
$180_noname	149	218	2520
$181_noname	72	147	2589
$182_noname	197	386	2475
$183_noname	52	136	2664
$184_noname	111	45	2748
$185_noname	113	73	3765
$186_noname	279	116	3725
$187_noname	71	41	3562
$188_noname	66	39	3532
$189_noname	31	122	3414
$190_noname	88	37	3505
$191_noname	134	108	3590
$192_noname	109	78	3564
$193_noname	142	63	3533
$194_noname	628	178	3454

$195_noname	338	387	2955
$196_noname	214	138	3004
$197_noname	234	57	2928
$198_noname	288	436	2475
$199_noname	130	258	2623
$200_noname	216	104	2751
$201_noname	143	468	2314
$202_noname	99	196	2639
$203_noname	145	153	2681
$204_noname	161	209	2718
$205_noname	247	157	2766
$206_noname	99	112	2676
$207_noname	270	317	2689
$208_noname	73	76	2736
$209_noname	85	112	3584
$210_noname	167	386	3166
$211_noname	129	355	3385
$212_noname	123	72	3611
$213_noname	151	82	3360
$214_noname	197	142	3291
$215_noname	264	67	3416
$216_noname	201	174	3219
$217_noname	426	153	3434
$218_noname	48	79	3161
$219_noname	254	265	3192
$220_noname	70	103	3203
$221_noname	195	103	3236
$222_noname	81	339	3144
$223_noname	96	254	3402
$224_noname	74	63	3560
$225_noname	688	245	3839
$226_noname	94	96	3394
$227_noname	389	351	3396
$228_noname	196	92	3591
$229_noname	391	262	3487
$230_noname	101	294	3358
$231_noname	77	271	3046
$232_noname	55	208	3087
$233_noname	39	168	3240
$234_noname	70	252	3369
$235_noname	91	89	3551
$236_noname	202	56	3549
$237_noname	84	107	3507
$238_noname	427	300	3530
$239_noname	372	41	3403
$240_noname	366	68	3072
$241_noname	334	472	2636
$242_noname	251	139	2774
$243_noname	137	162	2662
$244_noname	74	126	2687

$245_noname	102	67	2739
$246_noname	503	732	2108
$247_noname	51	418	2337
$248_noname	53	31	2704
$249_noname	210	19	2682
$250_noname	570	44	3017
*$251_noname	1374	32	2491
$252_noname	608	135	1149

*251_noname is the node representing Halobcateria Last Common Ancestor
